# Discovery of Sexual Dimorphisms in Metabolic and Genetic Biomarkers

**DOI:** 10.1371/journal.pgen.1002215

**Published:** 2011-08-11

**Authors:** Kirstin Mittelstrass, Janina S. Ried, Zhonghao Yu, Jan Krumsiek, Christian Gieger, Cornelia Prehn, Werner Roemisch-Margl, Alexey Polonikov, Annette Peters, Fabian J. Theis, Thomas Meitinger, Florian Kronenberg, Stephan Weidinger, Heinz Erich Wichmann, Karsten Suhre, Rui Wang-Sattler, Jerzy Adamski, Thomas Illig

**Affiliations:** 1 Unit of Molecular Epidemiology, Helmholtz Center Munich, German Research Center for Environmental Health, Neuherberg, Germany; 2 Institute of Genetic Epidemiology, Helmholtz Center Munich, German Research Center for Environmental Health, Neuherberg, Germany; 3 Institute of Bioinformatics and Systems Biology, Helmholtz Center Munich, German Research Center for Environmental Health, Neuherberg, Germany; 4 Institute of Experimental Genetics, Genome Analysis Center, Helmholtz Center Munich, German Research Center for Environmental Health, Neuherberg, Germany; 5 Department of Biology, Medical Genetics, and Ecology, Kursk State Medical University, Kursk, Russia; 6 Institute of Epidemiology II, Helmholtz Center Munich, German Research Center for Environmental Health, Neuherberg, Germany; 7 Institute of Human Genetics, Helmholtz Center Munich, German Research Center for Environmental Health, Neuherberg, Germany; 8 Institute of Human Genetics, Klinikum Rechts der Isar, Technische Universität München, Munich, Germany; 9 Division of Genetic Epidemiology, Department of Medical Genetics and Molecular and Clinical Pharmacology, Innsbruck Medical University, Innsbruck, Austria; 10 Department of Dermatology, Venereology, and Allergy, University Hospital Schleswig-Holstein, Kiel, Germay; 11 Institute of Epidemiology I, Helmholtz Center Munich, German Research Center for Environmental Health, Neuherberg, Germany; 12 Institute of Medical Informatics, Biometry, and Epidemiology, Chair of Epidemiology, Ludwig-Maximilians-Universität, Munich, Germany; 13 Klinikum Grosshadern, Munich, Germany; 14 Faculty of Biology, Ludwig-Maximilians-Universität, Planegg-Martinsried, Germany; 15 Weill Cornell Medical College in Qatar, Qatar Foundation, Education City, Doha, Qatar; 16 Lehrstuhl für Experimentelle Genetik, Technische Universität München, Munich, Germany; University of Oxford, United Kingdom

## Abstract

Metabolomic profiling and the integration of whole-genome genetic association data has proven to be a powerful tool to comprehensively explore gene regulatory networks and to investigate the effects of genetic variation at the molecular level. Serum metabolite concentrations allow a direct readout of biological processes, and association of specific metabolomic signatures with complex diseases such as Alzheimer's disease and cardiovascular and metabolic disorders has been shown. There are well-known correlations between sex and the incidence, prevalence, age of onset, symptoms, and severity of a disease, as well as the reaction to drugs. However, most of the studies published so far did not consider the role of sexual dimorphism and did not analyse their data stratified by gender. This study investigated sex-specific differences of serum metabolite concentrations and their underlying genetic determination. For discovery and replication we used more than 3,300 independent individuals from KORA F3 and F4 with metabolite measurements of 131 metabolites, including amino acids, phosphatidylcholines, sphingomyelins, acylcarnitines, and C6-sugars. A linear regression approach revealed significant concentration differences between males and females for 102 out of 131 metabolites (*p-values*<3.8×10^−4^; Bonferroni-corrected threshold). Sex-specific genome-wide association studies (GWAS) showed genome-wide significant differences in beta-estimates for SNPs in the *CPS1* locus (carbamoyl-phosphate synthase 1, significance level: p<3.8×10^−10^; Bonferroni-corrected threshold) for glycine. We showed that the metabolite profiles of males and females are significantly different and, furthermore, that specific genetic variants in metabolism-related genes depict sexual dimorphism. Our study provides new important insights into sex-specific differences of cell regulatory processes and underscores that studies should consider sex-specific effects in design and interpretation.

## Author Summary

The combination of genomic and metabolic studies during the last years has provided astonishing results. However, most of the studies published so far did not consider the role of sexual dimorphism and did not analyse their data stratified by sex. The investigation of 131 serum metabolite concentrations of >3,300 population-based samples (KORA F3/F4) revealed significant differences in the metabolite profile of males and females. Furthermore, a genome-wide picture of sex-specific genetic variations in human metabolism (>2,000 subjects from KORA F3/F4 cohorts) was investigated. Sex-specific genome-wide association studies (GWAS) showed differences in the effect of genetic variations on metabolites in men and women. SNPs in the *CPS1* (carbamoyl-phosphate synthase 1) locus showed genome-wide significant differences in beta-estimates of sex-specific association analysis (significance level: 3.8×10^−10^) for glycine. As global metabolomic techniques are more and more refined to identify more compounds in single biological samples, the predictive power of this new technology will greatly increase. This suggests that metabolites, which may be used as predictive biomarkers to indicate the presence or severity of a disease, have to be used selectively depending on sex.

## Introduction

Metabolomics provides a powerful tool to analyse physiological and disease-induced biological states on the molecular level, taking into account both the organism's intrinsic properties, i.e. genetic factors, and the effects of lifestyle, diet, and environment. The development of sophisticated analytic platforms and modern computational tools to handle increasingly complex data now enables the quantification of hundreds of metabolites from complex biological samples with a high throughput rate. These advancements support the integration of metabolomic profiles with genetic, epigenetic, transcriptomic and proteomic data for holistic systems biology approaches. Recently, common genetic variants have been demonstrated to exert large effects on individual metabolic capacities called “genetically determined metabotypes” [1], [2]. Therefore genetic variants in metabolism-related genes led to specific and clearly differentiated metabolic phenotypes [1], [3]. Knowledge on such genetically determined metabotypes is of crucial importance to understand the contribution and complex interaction of genes, proteins and metabolites in health and disease. Consequently, genetic studies can help to elucidate the direction of effects between metabolites and a specific disease. Thus, the combination of genetic and metabolic markers is an important emerging approach for biological research. To uncover potentially confounding influences on the interpretation of metabolic results, it is important to minimize the occurring confounders on human serum metabolites in a population-based study that has not been subjected to lifestyle and dietary controls. Pointed out recently, gender inequalities are another increasingly recognized problem in both basic research and clinical medicine [4]. Nevertheless, many published studies did not analyse their data stratified by sex [4]–[6] although there is a strong correlation between sex and the incidence, prevalence, age at onset, symptoms and severity of a disease, as well as the reaction to drugs [7], [8]. A survey of studies published in 2004 of nine different medical journals found that only 37% of participants were women (24% when restricted to drug trials), and only 13% of studies analysed data by sex [4]. Therefore we systematically assessed the effect of sex on serum metabolites in a large population-based cohort [9]. Furthermore, we investigated whether there are sex-specific differences in the genetic determination of metabotypes.

## Results

### Phenotypic Metabotype Differences between Males and Females

All phenotypic analysis steps were performed on population-based cohort data from KORA F4 (1452 males, 1552 females) and KORA F3 (197 males, 180 females, Figure S1) with fasting serum concentrations of 131 metabolites. The metabolites covered a biologically relevant panel that could be divided into five subgroups such as amino acids, sugars, acylcarnitines and phospholipids. Further information concerning the study population, sampling methods and the metabolite panel are described in the Material and Methods section and in the Tables S1, S6, and S7 and Figure S3.

**Figure 1:**
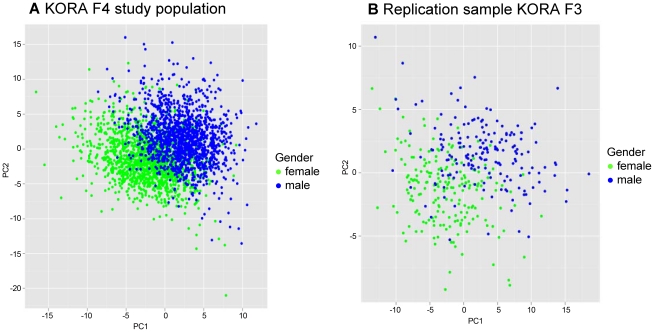
Two dimensional partial least square (PLS) analyses showing the contribution of 131 metabolites in males and females.

A Partial Least Square (PLS) analysis [10] of all metabolites showed that there were major differences of mean serum metabolite concentrations between males and females, as the values for the first two PLS components clustered clearly for men and women (Figure 1).

Motivated by the global gender differences in metabolite concentrations shown by PLS analysis, furthermore, the effect of sex on each metabolite was analysed using linear regression analysis. For each metabolite we calculated a linear regression with the logarithm of the metabolite concentration as dependent and sex as explanatory variable besides the covariates age, BMI and internal batch. The test whether the explanatory variable sex has a significant effect on the logarithm of the metabolite concentration revealed significant effects of gender in 102 of 131 metabolites (*p-value* below the Bonferroni-corrected significance level of 3.8×10^−4^). At least one metabolite of each subgroup including amino acids, acylcarnitines, phosphatidylcholines, lysophosphatidylcholines and sphingomyelins showed significant sex-specific differences in metabolite concentrations. In Table 1 the results of the linear regression analysis for representatives of each subgroup are presented, the complete list of results can be found in the Table S2.

**Table 1:** Phenotypic metabotype differences between males and females of the discovery set (KORA F4) and the replication study (KORA F3).

	Discovery			Replication			Metaanalysis	
metabolites	ß	*p-value*	r^2^	ß	*p-value*	r^2^	ß	*p-value*
**acylcarnitines**								
C18	0.146	5.6E-57	21.1%	0.092	3.6E-04	8.4%	0.140	2.5E-61
C10	0.089	2.3E-10	7.9%	0.068	1.0E-01	7.4%	0.087	5.8E-11
C10.1	0.088	5.2E-14	15.9%	0.061	1.0E-01	10.2%	0.085	1.3E-14
**amino acids**								
xLeu	0.206	1.6E-190	30.2%	0.165	1.1E-15	22.9%	0.202	3.8E-235
Val	0.142	1.9E-78	23.9%	0.096	2.4E-07	18.6%	0.136	5.4E-88
Gly	−0.130	9.1E-46	10.9%	−0.112	2.4E-06	11.1%	−0.128	3.4E-52
**phosphatidylcholines**								
PC aa C32∶3	−0.192	1.4E-106	15.6%	−0.272	1.4E-23	24.5%	−0.200	1.3E-138
PC aa C28∶1	−0.133	1.1E-53	8.5%	−0.219	4.7E-18	18.8%	−0.143	1.8E-71
PC ae C40∶3	−0.160	5.0E-99	18.7%	−0.177	2.6E-14	16.0%	−0.161	3.0E-120
PC ae C30∶2	−0.152	9.1E-53	8.1%	−0.214	1.1E-22	22.8%	−0.164	4.2E-77
**lysophosphatidylcholines**								
lysoPC a C20∶4	0.191	5.4E-62	10.8%	0.125	9.7E-05	8.6%	0.184	2.1E-67
lysoPC a C18∶2	0.183	6.2E-55	22.6%	0.136	4.7E-05	17.6%	0.178	1.8E-60
lysoPC a C18∶1	0.145	1.4E-41	12.7%	0.106	1.9E-04	16.3%	0.140	1.5E-45
**sphingomyelins**								
SM (OH) C22∶2	−0.228	1.1E-124	19.6%	−0.274	3.5E-25	27.3%	−0.234	1.7E-163
SM C18∶1	−0.200	1.3E-101	20.1%	−0.266	3.4E-26	27.0%	−0.209	1.1E-136
SM C20∶2	−0.283	7.5E-100	17.7%	−0.280	6.8E-26	25.8%	−0.282	1.0E-135
**hexoses**								
H1	0.065	6.2E-27	10.5%	0.029	1.6E-01	7.4%	0.062	3.0E-27

*P-values* were calculated by a linear regression model with metabolites as dependent variable and sex as explanatory variable adjusted for age and BMI. Presented is a set of results of highly significant metabolite concentration differences between males and females of each metabolite subclass out of the 131 tested metabolites. A full list of results for all metabolites and additional information on the complete metabolite panel is provided as supplementary data (Table S2 and S3). Significance level after Bonferroni-correction is *p-value*  =  3.8×10^-4^.

C5  =  valerylcarnitine, C0  =  carnitine, C18  =  octadecanoylcarnitine, xLeu  =  isoleucine+leucine, Val  =  valerine, Gly = glycine, PC aa Cx∶y  =  phosphatidylcholine diacyl x∶y, PC ae Cx∶y  =  phosphatidylcholine acyl-akyl Cx∶y, LysoPC a Cx∶y  =  lysophosphatidylcholine acyl Cx∶y, SM (OH) Cx∶y  =  hydroxyshingomyeline Cx∶y, SM Cx∶y  =  shingomyelin Cx∶y; ß  =  beta-estimate of linear regression, r^2^  =  explained variance.

**Figure 2:**
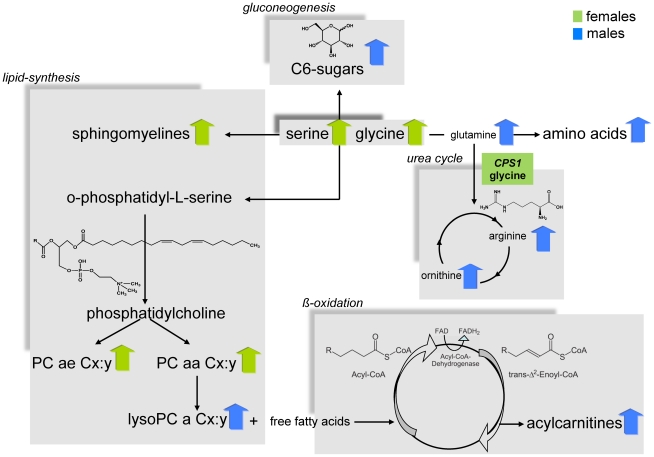
Systematic view of metabotype variations in the metabolism of males and females. It also shows the suggestive locus that is located in a gene encoding an enzyme that is central in human metabolism. *CPS1* is related to the amino acid metabolism. For this locus the metabolite with the strongest association is provided (green box). A blue arrow indicates metabolite concentrations which are higher in men than in women; green arrows vice versa.

The linear regression analysis showed that the concentrations of most amino acids were significantly higher in males except for the concentrations of glycine (effect of sex: ß = −0.13, *p-value* = 2.3×10^−46^) and serine (effect of sex: ß = −0.13, *p-value* = 1.0×10^−12^) which displayed higher concentrations in females. (Table 1, Table S2). The relative sex-specific difference for glycine was Δ = −14% (Table S8). That means that the mean concentration in men was 14% lower than in women (see Material and Methods). The levels of most serum acylcarnitines were significantly higher in males compared to females (Table 1, Table S2 and S8). The concentrations of phosphatidylcholines (PC ae Cx∶y or PC aa Cx∶y) tended to be significantly lower in males compared to females. The most significant difference between gender could be seen for the phosphatidylcholine PC aa C32∶3 (Δ = −17.9%, *p-value* = 4.4×10^−108^), whereas lysophosphatidylcholine (lysoPC a Cx∶y) concentrations were higher in males compared to females. In contrast, the concentrations of most sphingomyelins were significantly lower in men than in women (Table 1, Table S2 and S8). The concentration of H1 which is the sum of C6-sugars, was significantly higher in males compared to females (Δ = 7.3%, *p-value* = 6.2×10^−27^) (Table 1, Table S2 and S8). Figure 2 systematically reviews the sex-specific metabolite variations identified in this study.

The adjustment for different covariates (e.g.: waist-hip ratio (WHR), HDL (high density lipoprotein), LDL (low density lipoprotein), triglycerides, type 2 diabetes, smoking and high alcohol consumption) did not affect the sex-specific differences in the metabolite concentrations extensively. The majority of the high significant sex-effects remained significant. In particular, adjustments for lipid parameter (HDL, LDL and triglycerides), type 2 diabetes, smoking and high alcohol consumption did not influence our main findings. If WHR was included into the linear regression model as covariate replacing BMI or as additional covariate besides BMI, the *p-values* of the sex-effect on metabolites changed, but for most metabolite concentrations the gender-differences remained significant. Interestingly, there were seven PC aa Cx∶ys and Lyso PC a C17∶0 that showed significant differences between sexes while adjusting for age and WHR but not for BMI and age adjustment. We refer the interested reader to Table S2.

As replication the same linear regression approach (covariates: age, BMI) was applied to the KORA F3 cohort which included 377 individuals (Figure S1). Despite this smaller sample size for 63 of 102 metabolites with a significant effect of sex in KORA F4, the effect of sex in KORA F3 had the same direction and a significant *p-value* lower than the Bonferroni-corrected replication significance level corrected for the 102 metabolites taken forward to replication (0.05/102 = 4.9×10^−4^). That means 61.8% of the sex-specific differences could be replicated (Table 1, Table S3).

A combined meta-analysis of KORA F4 and KORA F3 revealed 113 metabolites with a significant effect of sex (Bonferroni-corrected meta-analysis significance level: p<3.8×10^−4^) (Table S3).

### Sex-Specific Effects in the Metabolic Network

**Figure 3:**
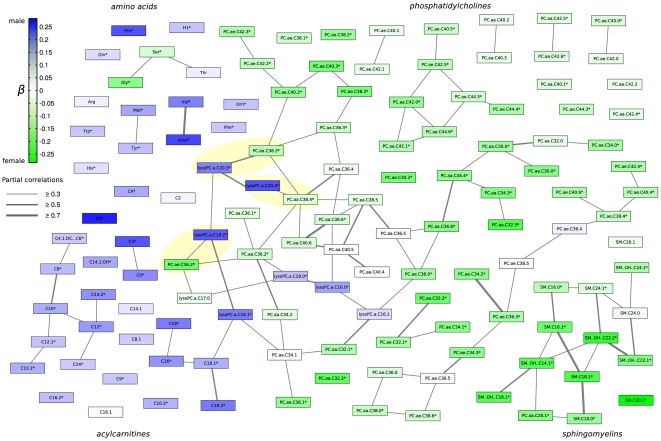
Gaussian graphical model of all measured metabolites illustrating the correlation strength and the propagation of gender-specific effects through the underlying metabolic network. Each node represents one metabolite whereas edge weights correspond to the strength of partial correlation. Only edges with a partial correlation above r =  0.3 are shown. Node colouring represents the strength of association (measured using β from linear regression analysis) towards either males or females. Metabolite names marked with a star * represent significantly different metabolites between genders. Yellow highlighted pairs of metabolites differ by a C18∶0 fatty acid residue.

We further investigated how groups of metabolites share pairwise correlations, that mean similar effects, and how the sex-specific effects propagate through the metabolic network. Therefore we calculated a partial correlation matrix between all metabolites, corrected against age, sex and BMI [11]. The resulting network, which is also referred to as a Gaussian graphical model (see Material and Methods), was annotated with the results from the linear regression analysis to get a comprehensive picture of sex-effects in this data-driven metabolic network (Figure 3). We applied a cut-off of r = 0.3 (r = partial correlation coefficient) in order to emphasize strong inter-metabolite effects. We observe a general structuring of the network into members from similar metabolic classes, e.g. the amino acids, the phosphatidylcholines, sphingomyelins and acylcarnitines (Figure 3). Direct correlations between metabolites, as represented by partial correlation coefficients, are rare in this metabolite panel with only around 1% of all partial correlations showing a strong effect above r = 0.3 (Figure S4 and S5). For this specific cut-off we obtained 14 non-singleton groups, which can be regarded as independently regulated phenotypes within the measured metabolite panel. Detailed description of the distribution of partial correlations and the group structure in the network can be found in Figure S4 and S5. The low connectedness of the network is in line with findings from *Krumsiek et al. 2011* [11] who demonstrated that Gaussian graphical models are sparsely connected on the one hand, but specifically exclude indirect metabolic interactions on the other hand.

Strikingly, sex-specific effects appear to be localized with respect to metabolic classes and connections in the partial correlation matrix. For instance, while most sphingomyelin concentrations have been shown to be higher in females, we also observe them to be a connected component in the GGM. Similarly, acylcarnitines are higher in males and also share partial correlation edges, mostly with other acylcarnitines (Figure 3). Interestingly, we observed three metabolite pairs from the PC aa and lyso-PC classes, respectively, which constitute a side chain length difference of 18 carbon atoms (yellow shaded metabolite pairs, Figure 3).

### Genotypic Metabotype Differences between Males and Females

For the identification of differences in genetically determined metabotypes, we used a subpopulation of 1809 participants of the KORA F4 (Geno-KORA F4) study. The replication was done in an independent subsample of KORA F4 (Rep-KORA F4) and in a subsample of the KORA F3 (Rep-KORA F3) cohort, see Material and Methods for details and Figure S1.

**Figure 4:**
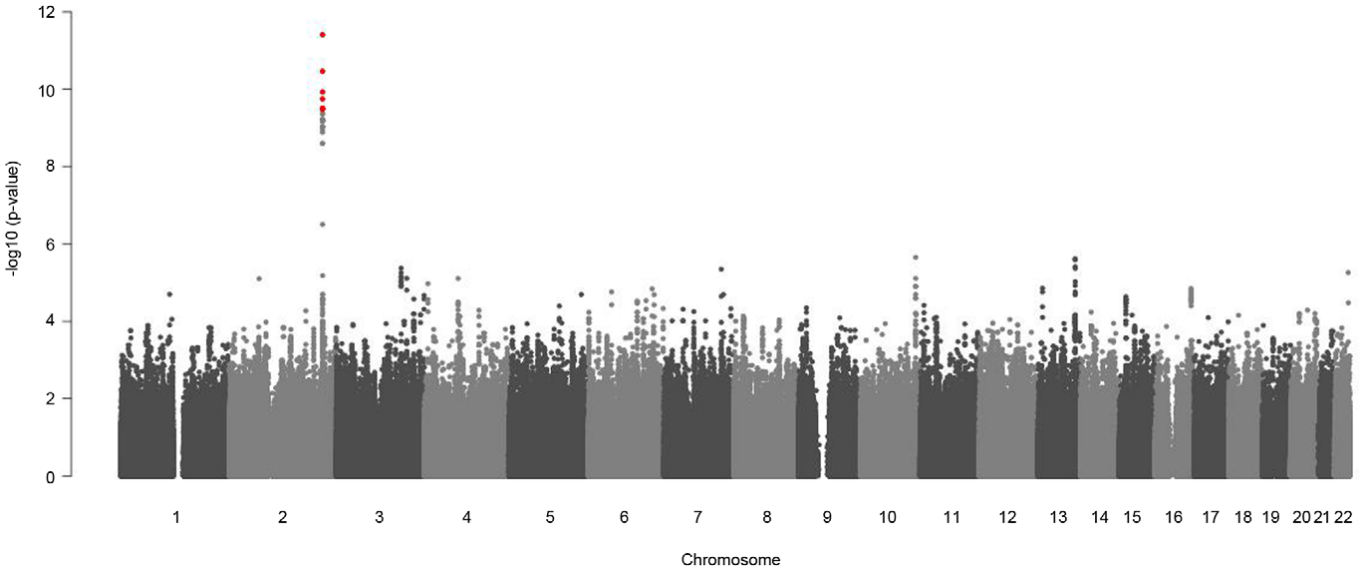
Manhattan plots for gender-specific genome-wide beta-differences for the metabolite glycine. Genome-wide significant beta-differences are plotted in red (significance level p<3.8×10^−10^).

Sex-stratified genome-wide association analysis adjusted for age and BMI were performed for logarithmized concentrations of all metabolites. In order to reveal gender differences we tested the estimated SNP effects for heterogeneity between men and women (see Material and Methods, Figure 4 and Figure S2). We applied a Bonferroni-corrected genome-wide significance level of 5×10^−8^/131 = 3.8×10^−10^. All SNPs with a minor allele frequency (maf) lower than 1% in men or in women were excluded. Moreover, SNPs with a low quality of imputation (rsq<0.4) were also excluded. Eight SNPs on chromosome 2 showed genome-wide significant differences in SNP effects (beta-estimates) between men and women for association with glycine (Table 2). The absolute beta-estimates of all eight significant SNPs were higher in women compared to men. The strongest gender difference was seen at SNP rs715 with a genome-wide significant *p-value* of 3.65×10^−10^ for the test of beta-estimate differences. For men the observed effect of rs715 was -0.067 and for women −0.2 (Table 2). SNP rs715 is part of the 3′ UTR region of the *CPS1* gene. SNP rs7422339 with a *p-value* of 3.24×10^−11^ for the test of beta-estimate differences is in a non synonymous coding region of *CPS1*. All other significant SNPs are intergenetic but located in the same region (Table S5). Local association plots for the association of this region with glycine for males and females are presented in Figure S3. The differences in beta-estimates remained significant for GWAs with adjustment for WHR instead of BMI or BMI and WHR combined (Table S4).

**Table 2:** List of SNPs with significant differences in beta-estimates between men and women for association with glycine observed in Geno-KORA F4.

		Geno-KORA F4			Rep-KORA F4			Rep-KORA F3			combined		
SNP	effect allele	effect men	effect women	pval (beta diff)	effect men	effect women	pval (beta diff)	effect men	effect women	pval (beta diff)	effect men	effect women	pval (beta diff)
rs715	T	−0.067± (0.012)	−0.206± (0.016)	3.65E-12	-	-	-	-	-	-	−0.067± (0.012)	−0.206± (0.016)	3.65E-12
rs7422339	C	−0.078± (0.013)	−0.22± (0.017)	3.24E-11	−0.081± (0.012)	−0.225± (0.015)	1.30E-13	−0.115± (0.031)	−0.229± (0.043)	0.03151	−0.082± (0.009)	−0.223± (0.011)	2.12E-24
rs10172053	T	−0.043± (0.012)	−0.172± (0.016)	1.12E-10	-	-	-	−0.113± (0.033)	−0.113± (0.047)	1	−0.051± (0.011)	−0.166± (0.015)	1.19E-09
rs7424145	G	−0.041± (0.011)	−0.165± (0.016)	1.70E-10	-	-	-	−0.109± (0.032)	−0.161± (0.045)	0.34633	−0.048± (0.01)	−0.165± (0.015)	2.17E-10
rs10490325	G	0.043± (0.012)	0.169± (0.016)	2.98E-10	-	-	-	0.107± (0.032)	0.133± (0.045)	0.63774	0.051± (0.011)	0.165± (0.015)	1.29E-09
rs2160847	T	−0.036± (0.012)	−0.162± (0.016)	2.98E-10	-	-	-	−0.114± (0.034)	−0.126± (0.046)	0.83384	−0.045± (0.011)	−0.158± (0.015)	1.77E-09
rs2216405	G	0.043± (0.012)	0.169± (0.016)	2.98E-10	-	-	-	0.107± (0.032)	0.127± (0.045)	0.7172	0.051± (0.011)	0.164± (0.015)	1.62E-09
rs4673546	T	0.038± (0.011)	0.155± (0.015)	3.18E-10	-	-	-	0.105± (0.031)	0.149± (0.044)	0.41365	0.046± (0.01)	0.154± (0.014)	6.13E-10

Replication results for these SNPs in Rep-KORA F4 and Rep-KORA F3 are also presented. Not all SNPs were available for all replication cohorts, because different genotyping and imputation methods were used. For the combined analysis the sex-specific effects of all three studies are metaanalyzed and the beta-difference is calculated based on these sex-specific meta-analysis beta-estimates.

The significant differences in beta-estimates between men and women for the association of the eight reported SNPs with glycine were replicated in two independent cohorts Rep-KORA F4 and Rep-KORA F3, including 788 women and 758 men. In the first replication cohort Rep-KORA F4 (583 men, 635 women) the absolute beta-estimate for SNP rs7422339 was also higher in women (beta = −0.225) compared to men (beta = −0.081). The absolute difference of the beta-estimates for SNP rs7422339 was with 0.144 similar to the difference observed in the discovery sample. The *p-value* of the test for difference in beta-estimates of the replication was 1.3×10^−13^. The other seven SNPs were not available for the Rep-KORA F4 cohort due to other genotyping methods. In the second but smaller Rep-KORA F3 cohort seven of the eight SNPs were available. For SNP rs7422339 we observed that the absolute beta-estimate in men (beta = −0.115) was also lower than the absolute beta-estimate in women (beta  =  −0.229). The absolute difference of the beta-estimates for SNP rs7422339 was 0.144 similar to the absolute difference in beta-estimates observed in the discovery and the first replication cohort (Rep-KORA F4). The *p-value* for the test of difference in beta-estimates was not significant in Rep-KORA F3 (*p-value* = 0.032). For the remaining six SNPs, which were taken forward for replication in Rep-KORA F3 the beta-estimates were also lower in males compared to females but the *p-values* of the test of beta-differences between men and women were not significantly replicated in the Rep-KORA F3 cohort (Table 2).

## Discussion

There have been only few studies addressing metabolic differences between males and females, and most of these studies were rather small in sample size and determined only a small number of metabolites [5], [12]. We investigated a large population-based study with sufficient statistical power to examine associations within subgroups and a large number of metabolites. Our findings shed light on sex-specific architecture of the human metabolome and provide clues on biochemical mechanisms that might explain observed differences in susceptibility and time course of the development of common diseases in males and females. Our data provided new insights into sex-specific metabotype differences. Combining results from linear regression with partial correlation analysis (resulting in a Gaussian graphical model) yielded interesting insights into how sex-specific concentration differences spread over the metabolic network (Figure 3). The analysis suggests that sex-specific concentration differences affect whole metabolic pathways rather than being randomly spread over the different metabolites. In addition, we found three interesting inter-class associations between PCaa/PCae species and lyso PC species (highlighted in yellow in Figure 3). Those pairs shared a strong partial correlation but displayed differential concentration patterns with respect to gender effects. Furthermore, these pairs displayed a fatty acid residue difference of C18∶0, indicating that this fatty acid species might be a key compound giving rise to opposing metabolic gender effects.

Direct experimental evidence indicated a role for sphingolipids (sphingomyelins and ceramides) in several common complex chronic disease processes including atherosclerotic plaque formation, myocardial infarction (MI), cardiomyopathy, pancreatic beta cell failure, insulin resistance, coronary heart disease and type 2 diabetes (T2D) [13], [14]. Already young children (between birth and 4 years old, with low levels of sex-hormones) may reveal significant sex-specific differences in plasma sphingolipid concentrations [15]. Our observations described new sex-specific differences, while other lipid-derived molecules, like bile acids, were already demonstrated not to be sex-specific [16]. Therefore sphingomyelins represent important intermediate phenotypes. The concentration differences between males and females of acylcarnitines described in this study coincide with previous findings showing that carnitine (C0) and acetylcarnitine (C2) concentrations were higher in males than in females[17], [18]. Phosphatidylcholines, as demonstrated in this study, are another gender-specific phenotype. Ghrelin (controlling energy homeostasis and pituitary hormone secretion in humans) levels have been shown to be similar in men and women and did not vary by menopausal status or in association with cortisol levels [19]. These findings of our and other studies urgently suggest when using metabolites for disease prediction gender has to be strictly taken into account.

A previously published non sex-stratified GWA study on metabolites based on the same population Geno-KORA F4 reported 15 loci which showed genome-wide significant associations with at least one metabolite concentration or ratio [1]. Besides others the locus *CPS1* was found to have a significant effect on glycine concentrations. But the findings of this sex-stratified genome-wide association analysis revealed that the genetic determination of *CPS1* differs significantly between males and females. Therefore it is important to analyse the data stratified by sex. SNP rs74223369 on chromosome 2 in the 3′ UTR region of the gene *CPS1* showed a genome-wide significant difference in beta-estimates between men and women for association with glycine. The gender-specific effect of SNP rs7422339 was significantly replicated as the difference between the beta-estimates of men and women was of the same direction in the discovery sample and in the replication cohort Rep-KORA F4 and the *p-value* of the test of differences was lower than the replication significance niveau (0.05/8). The other SNPs of the *CPS1* gene region also showed significant gender-specific effects but these effects could not be replicated in the Rep-KORA F3 cohort. As the effect-sizes and differences for the SNP rs7422339 are similar and at least for the other SNPs are pointing into the same direction as in the discovery set, the failed replication in Rep-KORA F3 might be a problem of power due to the smaller sample size.

*CPS1,* which encodes the mitochondrial enzyme *CPS1*, plays a pivotal role in protein and nitrogen metabolism catalyzing the first committed step of the hepatic urea cycle. Once ammonia has entered the mitochondria via glutamine or glutamate, CPS1 adds the ammonia to bicarbonate along with a phosphate group to form carbamoyl phosphate. Carbamoyl phosphate is then put into the urea cycle. The hepatic urea cycle is responsible for the elimination of ammonia in the form of urea as well as the synthesis of arginine. Among others, Döring *et al*. 2008 could show that there is strong evidence that, in addition to environmental components, a strong genetic and sex-specific control influences the regulation of blood uric acid concentration. They showed that the proportion of the variance of serum uric acid concentrations explained by *SLC2A9* genotypes was about 1.2% in men and 6% in women [12]. Brandstätter *et al.* 2010 also observed a sex-specific interaction with genetic association of atherogenic uric acid concentrations [20]. Paré *et al.* 2009 described that the *CPS1* SNP rs7422339, which encodes the substitution of asparagine to threonine (T1405N) in the region critical for N-acetyl-glutamate binding resulting in 20% to 30% higher enzymatic activity [21], is associated with homocysteine also in a sex-specific manner in their study [22]. Also in an Asian population the effects of the genetic variations of the *CPS1* gene were stronger in women than in men [23].

Interestingly a meta-analysis of genome-wide association data in 67,093 individuals also of European ancestry identified recently *CPS1* to affect creatinine production and secretion in Chronic Kidney Disease (CKD) [24]. Hicks *et al*. 2009 performed a GWAS of different circulating sphingolipids in five diverse European populations [3]. They could show associations of genetic loci with several lipid species but did not analyse their data stratified by sex. There is just some evidence for loci with differential sex-effects influencing classical lipids like HDL [25]. Therefore identification of sex-specific genetic variants, that alter the homeostasis of key metabolites in males and in females, will lead to a better functional understanding of the genetics of complex disorders.

As global ‘omics’-techniques are more and more refined to identify more compounds in single biological samples, the predictive power of these new technologies will greatly increase. Metabolite concentration profiles and genomic data can be used as predictive biomarkers to indicate the presence or severity of a disease depending on gender. Our study provides new important insights into sex-specific differences of cell regulatory processes and underscores that studies should consider gender-specific effects in design and interpretation. Our findings help to understand biochemical mechanisms underlying sexual dimorphism, a phenomenon which may explain the differential susceptibility to common diseases in males and females.

## Materials and Methods

### Ethics Statement

Written informed consent has been given by each participant. The study, including the protocols for subject recruitment and assessment and the informed consent for participants, was reviewed and approved by the local ethical committee (Bayerische Landesärztekammer).

### Study Population

The KORA S4 survey, an independent population-based sample from the general population living in the region of Augsburg, Southern Germany, was conducted in 1999/2001. The standardized examinations applied in the survey (4261 participants) have been described in detail elsewhere [1], [9], [26]. A total of 3080 subjects participated in a follow-up examination of S4 in 2006-08 (KORA F4), comprising individuals who, at that time, were aged 32–81 years (Figure S3). In a sample of 3061 individuals metabolomics data was available. This subgroup was used as discovery sample of the phenotypic analysis. For the GWAS the 3061 KORA F4 individuals with metabolomics measurements were divided into two subgroups. First, a subgroup of 1809 individuals from KORA F4 with, who were genotyped using a genome-wide SNP-array (see section genotyping and imputation): Geno-KORA F4. Second, a subgroup of 1218 individuals from KORA F4 with genotyping data generated by Metabo-Chip from Illumina: Rep-KORA F4. Since Geno-KORA F4 and Rep-KORA F4 are non overlapping subgroups of individuals from the KORA F4 cohort they can be considered independent. Therefore it was possible to take Geno-KORA F4 as discovery sample and Rep-KORA F4 as first replication sample for the GWAS.

The KORA F3 cohort is a ten years follow-up survey of the KORA S3 survey examined in 1994–1995 as described previously [26], [27]. For the replication step of the phenotypic analysis randomly selected 197 males and 180 females (aged 55–79 years) from the KORA F3 cohort were taken. For 328 individuals (175 males, 153 females) genome-wide genotypes were available. These were used as second replication cohort for the GWAS. No evidence of population stratification was found in multiple published analyses using the KORA cohort [28]. The KORA F3 and F4 surveys are completely independent with no overlap of individuals (Figure S3).

### Blood Sampling

Blood samples for metabolic analysis were collected during the years 2006 and 2008 in parallel with the KORA F4 examinations as described in [1], [2], and were deep frozen at −80°C until metabolomic analysis. To avoid variation due to circadian rhythm, the blood samples were drawn in the morning between 8:00 and 10:00 am after overnight fasting. Material was immediately horizontal shaken (10 min), followed by 40 min resting at 4°C to obtain complete coagulation. The material was then centrifuged (2000 g; 4°C). Serum was aliquoted and kept for 2–4 hours at 4°C, after which it was deep frozen to −80°C until sampling.

### Metabolite Measurements

Metabolomic analysis was performed on 3061 subjects from the population-based cohort KORA F4 and on 377 subjects of the population-based cohort KORA F3. Men and women were collected in a random order and samples were randomly put on plates to exclude batch effects.

Liquid handling of serum samples (10 µl) was performed with Hamilton Star (Hamilton Bonaduz AG, Bonaduz, Switzerland) robot and prepared for quantification using the *AbsoluteIDQ* kit (BIOCRATES Life Sciences AG, Innsbruck, Austria) as described previously [1]. Sample analysis were done on API 4000 QTrap LC/MS/MS System (Applied Biosystems, Darmstadt, Germany) equipped with Schimadzu Prominence LC20AD pump and SIL-20AC auto sampler. The complete analytical process was monitored with the *MetIQTM* software package, which is an integral part of the Absolute*IDQ™* kit.

### Metabolite Panel

In total, 163 different metabolites were quantified. More information about the metabolite panel can be found in Text S1. Metabolite measurements of the 3061 samples were performed in three batches, with two and three months time lapse in between, respectively. Within each kit, there are three different quality controls (QCs) representing gender mixed human plasma samples provided by the manufacturer. In accordance with the kit instructions, concentration of each metabolite was adjusted based on the three QCs to minimize the potential batch effects.

To ensure data quality, metabolites had to meet three criteria: (1) average value of coefficient of variance (CV) of the three QCs should be smaller than 25%. (2) 90% of all measured sample concentrations should be above the limit of detection (LOD). (3) Correlation coefficients between two duplicated measurements of 144 re-measured samples should be above 0.5 (Table S6). In total, 131 metabolites passed the three quality controls. To detect sample outliers, the data of the 131 metabolite concentrations were first scaled to zero mean and unity standard deviation and were projected onto the unit sphere and Mahalanobis distances were then obtained. Robust principal components algorithm was used in the process [29]. Mean and variance were then calculated for the distances. A cut-off was set at 3 times variance plus mean distance. Any individual, whose distance was greater than this cut-off, was marked as an outlier and removed. Outliers were detected separately for males and females. 131 Metabolites and 3004 samples remained in the dataset. Missing values were using the R package “mice”. Metabolite concentrations were logarithmized for all subsequent analysis steps.

### Genotyping and Imputation

In KORA F4 genome-wide genotyping was done using the Affymetrix 6.0 GeneChip array (Geno-KORA F4). The algorithm Birdseed2 was used for calling. Genotyped SNPs were filtered for an individual call rate of 0.93, SNP call rate 0.93 and Hardy-Weinberg equilibrium (*P*HWE >0.001). All remaining SNPs (651,596) were used for imputation with MACH (v1.0.15). HapMap CEU version 22 was used as reference population for calling and imputation. The GWAS replication cohort Rep-KORA F4 was genotyped on Metabo-Chip, with calling algorithm GenomeStudio. The second GWAS replication sample KORA F3 was genotyped with the Affymetrix 500 K array. The calling was performed by BRLMM with reference population HapMap CEU 21. After filtering for individual call rate 0.93 and SNP call rate 0.9 and Hardy-Weinberg equilibrium (*P*_HWE_ >0.001) the remaining SNPs were imputed with MACH v1.0.9 using HapMap CEU version 21 as reference population.

### Statistical Analysis

#### Partial least squares (PLS)

PLS, or projection to latent structures by means of partial least squares, and is a method to relate a matrix X to a vector y (or to a matrix Y).The x-data are transformed into a set of a few intermediate linear latent variables (components). PLS analysis [10] was carried out using the *R* package *pls* to investigate the metabolic profiles of males and females. Data was visualized by plotting the scores of the first two components against each other, where each point represented an individual serum sample. For this analysis, metabolite concentrations were normalized to have a mean of zero and a standard deviation of one.

#### Delta (difference in concentration means for men and women)

For comparison of metabolite concentrations between men and women we used the delta (Δ), as it describes the difference in concentration means for men and women for a specific metabolite relative to the mean metabolite concentration in men. Therefore the difference of mean metabolite concentration in men and mean metabolite concentration in women is calculated and than divided by the mean metabolite concentration in men. For example, a value of Δ = 50% means, that the mean metabolite concentration in women is 50% lower than the metabolite concentration in men.

#### Linear regression

Metabolite concentration differences between males and females were investigated by linear regression analysis. The basic model contains the log-transformed metabolite as dependent, sex as explanatory variable and both age and BMI as covariates. Moreover, an internal batch variable is included to account for possible systematic differences that might have been caused by the metabolite measuring process. To correct for multiple testing Bonferroni-correction was applied. That means the influence of sex on a specific metabolite was called significant, if the *p-value* of the corresponding test of sex having no effect on (log-transformed) metabolite is lower than 0.05/131 = 3.8×10^−4^. For replication we also applied Bonferroni-correction. That means a difference in sex on a specific metabolite is called significant, if the direction of the effect in consistent between discovery and replication cohort and the *p-value* for sex having no effect on the metabolite is lower than 0.05 corrected for the number of metabolites taken forward for replication.

We analysed the influence of anthropometric phenotypes, diseases and environmental factors by including different covariates to the linear regression and comparison of the structure of the results. Four models which differ in the use of one or more additional covariates were performed. The covariates in each model beside age are waist hip ratio (WHR), lipid parameters (HDL and LDL cholesterol, triglycerides), type 2 diabetes, alcohol consumption and smoking. All calculations were performed in R with standard procedures (lm). Furthermore, a meta-analysis of the discovery and the replication sample with a fixed effect model was analyzed to reveal the sex-specific effects of metabolite concentrations.

#### Partial correlation analysis

In order to investigate how strong the different metabolites correlate with each other and the sex-specific effects propagate through the underlying metabolic network, we calculated full-order partial correlation coefficients (r) between all pairs of metabolites. The resulting partial correlation networks are commonly referred to as Gaussian graphical models (GGMs), which we have previously demonstrated to be useful for the analysis of direct metabolite-metabolite effects in the same population cohort [11]. The GGM was coloured and annotated according to the β-values and *p-values* from linear regression analysis and then exported and visualized using the free yEd graph editor.

#### Genome-wide association studies (GWAS)

We calculated GWAS for all 131 metabolites with mach2qtl (v1.0.8) for men and women separately. We applied an additive model with covariates age, BMI and an internal variable accounting for batch effects.

#### Genome-wide test for sex-specific differences in beta-estimates

We tested each SNP and metabolite for equality of the beta-estimates for the SNP calculated in the sex-specific GWAS. Therefore we used an approximately normally distributed test statistic [30]: 
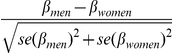



To take our 131 phenotypes into account we used Bonferroni correction. Therefore the genome-wide significance level is 5×10^−8^/131 = 3.8×10^−10^ [31].

#### Replication of sex-specific differences in genetic effects

We confirm a genetic sex-specific difference as replicated, if the proportion of the absolute SNP effects in men and women is the same as in the discovery sample, and the *p-value* for the test for difference in effects is lower than the adjusted *p-value*.

#### Replication in KORA F4

We used PLINK for the calculation.

#### Further analysis

All further analyses were performed in R. For both subgroups, men and women, we calculated the frequencies of each SNP. The explained variance (R^2^) for each SNP and metabolite was calculated as the difference of the coefficients of determination of the model with SNP and without SNP. The metaanalysis of SNP association with glycine was performed with METAL (http://www.sph.umich.edu/csg/abecasis/Metal/index.html) for men and women separately using the inverse variance weighting.

## Supporting Information

Figure S1 KORA study populations with subsamples used in this study.
(TIFF)

Figure S2 QQ-plots for the sex-stratified GWAS with glycine. The QQ-plot shows the p-values of the sex stratified GWAS for glycine in the discovery sample Geno-KORA F4 versus the expected *p-values* under the null hypotheses of no SNP having an effect on glycine.
(JPG)

Figure S3 Regional association plots for sex-stratified GWAS with glycine around the locus *CPS1*. Association *p-values* of SNPs with glycine for men and women are presented for a region surrounding rs715, which had the strongest difference in beta-estimates between men and women. SNPs with genome-wide significant differences in beta-estimates are highlighted in blue. The level of linkage disequilibrium of rs715 with other SNPs is indicated by circle colour ranging from red r^2^>0.8, orange 0.8>r^2^>0.5, grey 0.5>r^2^>0.2 to white 0.2>r^2^.
(TIFF)

Figure S4 Distribution of partial correlation coefficients. Partial correlations center around zero with a shift towards positive high values. When applying a correlation cutoff of r = 0.3, we are left with 109 out of 8515 correlation values (1.28%).
(TIFF)

Figure S5 Number of clustered groups in the GGM as a function of the absolute partial correlation cutoff. Note that we did not count singleton metabolites that is metabolites without any partial correlation above threshold, here. Most non-singleton groups emerge in the cutoff range between 0.3 and 0.7, which corresponds to the figure in the main manuscript. For our lower cutoff of 0.3, we obtain 14 groups, which can here be regarded as *independent phenotypes* in the metabolite pool.
(TIFF)

Table S1 Study population characteristics. Data are presented as mean (SD) or number of persons (N); BMI indicates body mass index; HDL high density lipoprotein; LDL low density lipoprotein; smokers: number of smokers with one or more than one cigarette/day, high alcohol intake: subjects were counted for high alcohol intake when they had an alcohol consumption of ≥0 g alcohol/day for males and ≥20 g alcohol/day for females. (A) Study populations used for phenotypic analysis. (B) Study populations used for genotypic analysis.
(DOCX)

Table S2 Phenotypic metabotype differences between males and females of the discovery sample KORA F4. *P*-values were calculated by a linear regression model with metabolite concentration as outcome and sex as explanatory variable adjusted for different covariables. Gray shaded columns show significant *p-values* for differences in the metabolite concentrations between males and females after Bonferroni correction (significance level after multiple testing correction * =  p-value*<3.8×10^−4^).
(DOCX)

Table S3 Phenotypic metabotype differences between males and females of the replication sample KORA F3. *P*-values were calculated by a linear regression model with metabolite concentration as outcome and sex as explanatory variable adjusted for age, BMI and waist-hip ratio (WHR). Gray shaded columns show significant *p-values* for differences in the metabolite concentrations between males and females after Bonferroni correction (significance level after multiple testing  =  *p-value*<3.8×10^−4^).
(DOCX)

Table S4 Comparison of different adjustments in association of SNPs with glycine. Results for SNPs which showed a significant difference in beta-estimates for KORA F4 with the adjustment of sex-specific GWAs for BMI (age, batch), for different adjustment for waist-hip ratio (WHR) (age, batch) or adjustment for WHR and BMI (age, batch).
(DOCX)

Table S5 Detailed information for SNPs with significant gender differences in beta-estimates for association with glycine. Minor allele frequency is calculated for men and women separately in each study. Imputation quality (RSQ) respectively call rate for genotyped SNPs is calculated based on all IDs.
(DOCX)

Table S6 Full biochemical names of all 131 metabolites used for further analysis that were measured on the Biocrates Absolute IDQ kit. Abbreviations and full biochemical names of the 131 metabolites are shown in the first and second columns, respectively. The third column lists shows the correlation coefficients (r) between two duplicated measurements of 144 re-measured samples. The following column shows percentage of 3061 individuals above limit of detection (LOD) and the last column shows the mean value of the correlation coefficient (CV) of the three quality controls for the three batches.
(DOCX)

Table S7 Excluded metabolites that were measured on the Biocrates AbsoluteIDQ kit. Abbreviations and full biochemical names of the excluded metabolites are shown in the first and second columns, respectively. The third column shows the correlation coefficients (r) between two duplicated measurements of 144 re-measured samples. The following column shows percentage of 3061 individuals above limit of detection (LOD). Mean value of the correlation coefficient (CV) of the three quality controls for the three batches is shown in the last column.
(DOCX)

Table S8 Metabolite concentrations of the study cohorts KORA F4 and KORA F3 and the relative sex-specific difference (Δ in %). Δ =  (Mean(metabolite concentration of men) – mean(metabolite concentration of women) ) /mean (metabolite concentration of men); difference of metabolite concentrations between men and women in %.
(DOCX)

Text S1 Metabolite panel.
(DOC)
