# Supplementary figures and images for: Discovery of Sexual Dimorphisms in Metabolic and Genetic Biomarkers
Source: PLoS Genet. 2011 Aug 11;7(8):e1002215. doi: 10.1371/journal.pgen.1002215 (PMC3154959; doi:10.1371/journal.pgen.1002215)

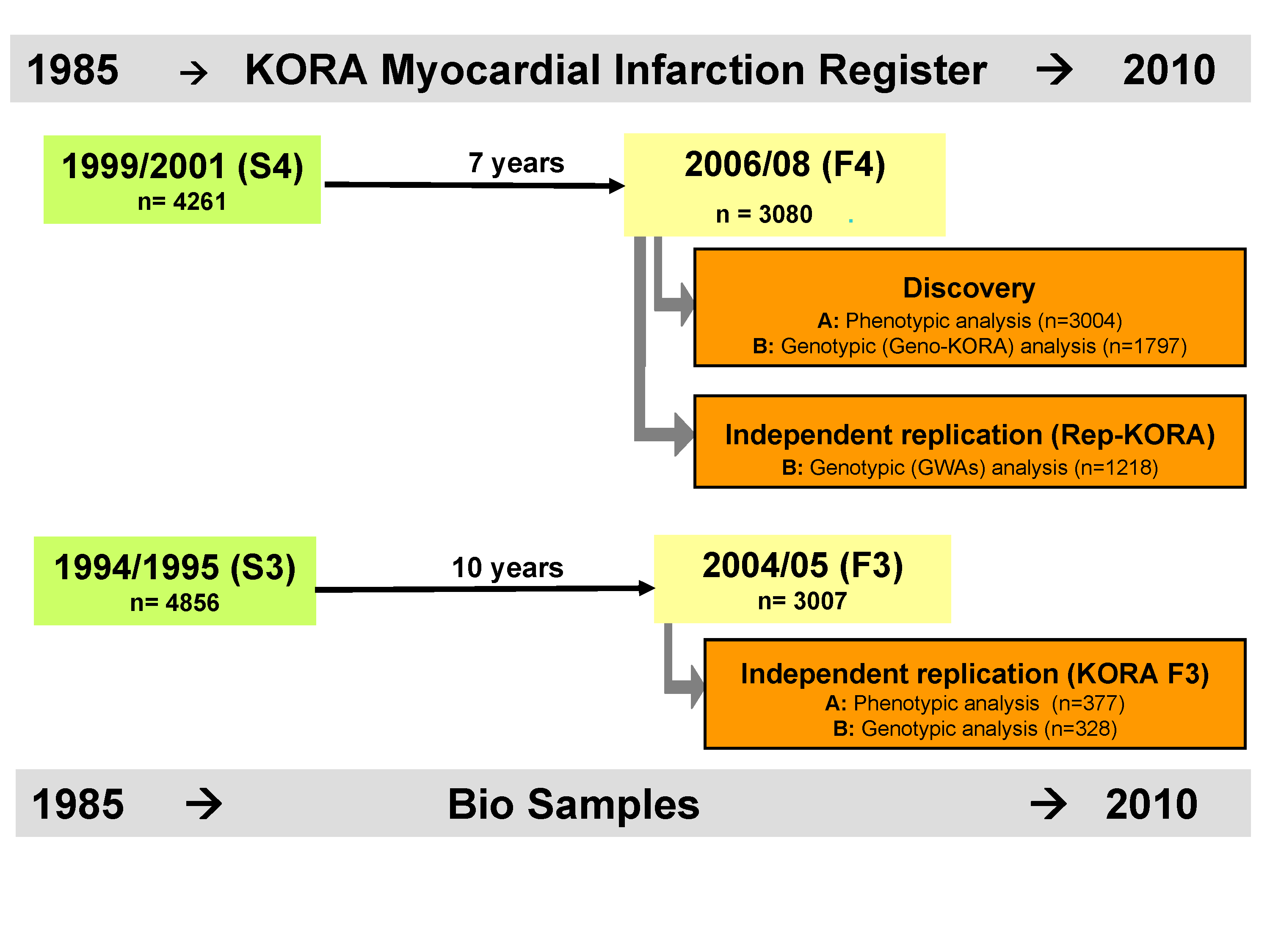

Supplement: Figure S1 — KORA study populations with subsamples used in this study. (TIFF) [file pgen.1002215.s001.tif]

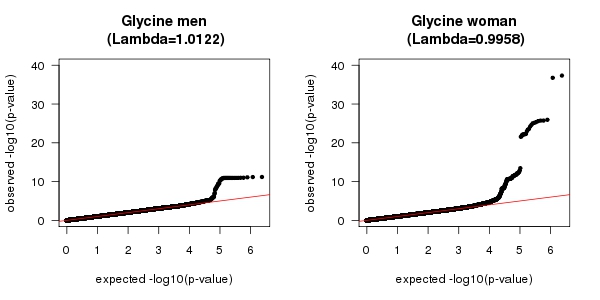

Supplement: Figure S2 — QQ-plots for the sex-stratified GWAS with glycine. The QQ-plot shows the p-values of the sex stratified GWAS for glycine in the discovery sample Geno-KORA F4 versus the expected p-values under the null hypotheses of no SNP having an effect on glycine. (JPG) [file pgen.1002215.s002.jpg]

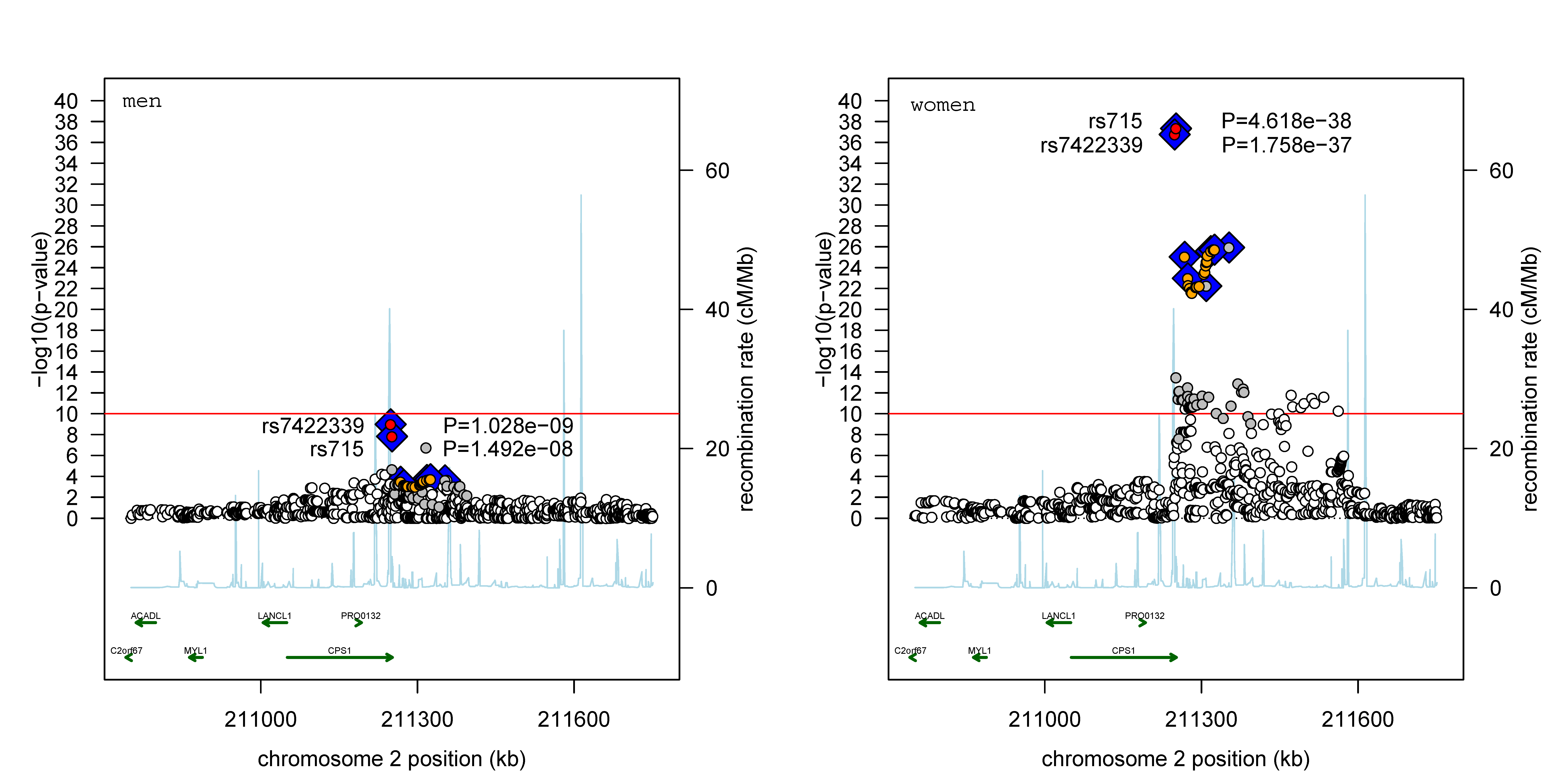

Supplement: Figure S3 — Regional association plots for sex-stratified GWAS with glycine around the locus CPS1. Association p-values of SNPs with glycine for men and women are presented for a region surrounding rs715, which had the strongest difference in beta-estimates between men and women. SNPs with genome-wide significant differences in beta-estimates are highlighted in blue. The level of linkage disequilibrium of rs715 with other SNPs is indicated by circle colour ranging from red r2>0.8, orange 0.8>r2>0.5, grey 0.5>r2>0.2 to white 0.2>r2. (TIFF) [file pgen.1002215.s003.tif]

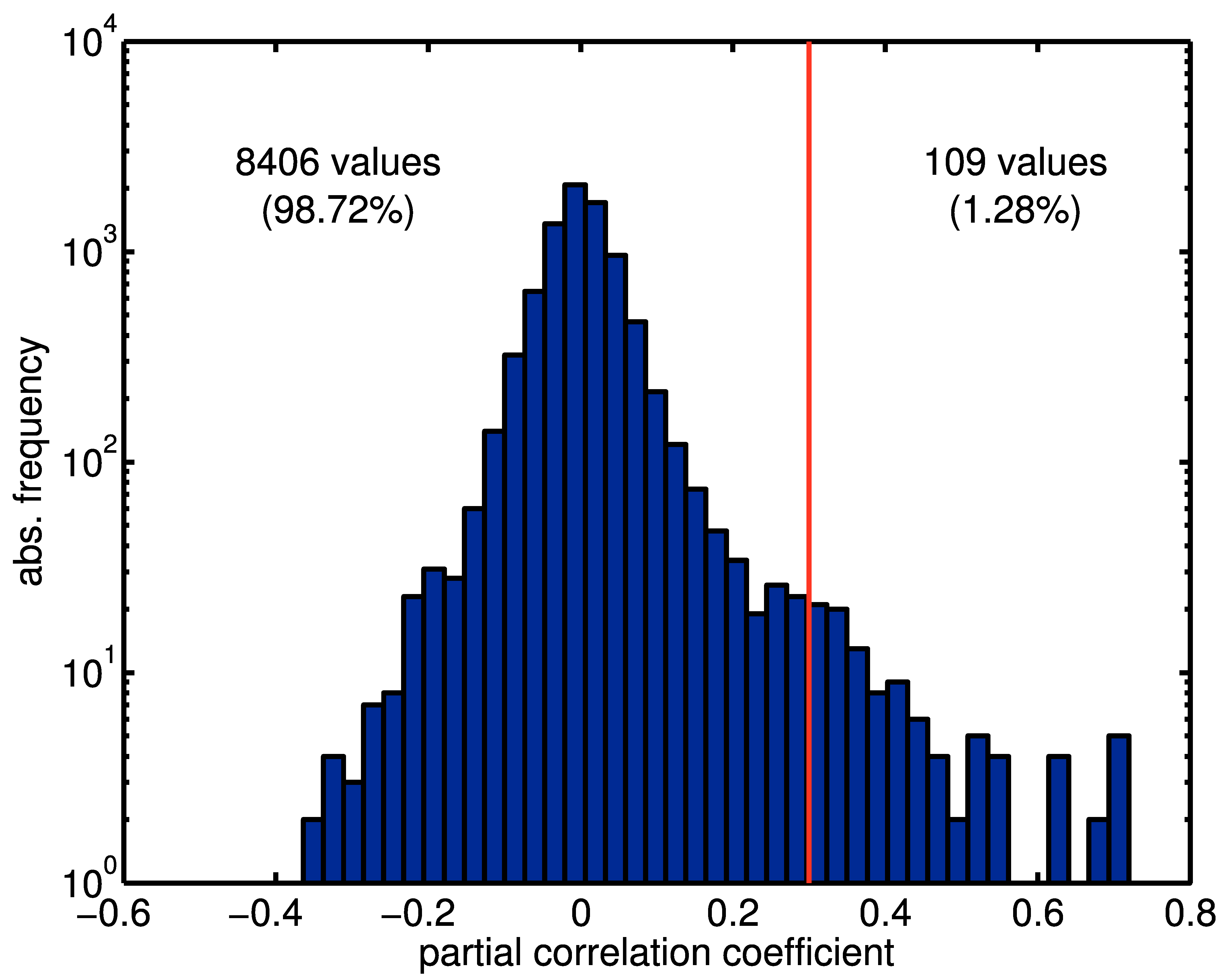

Supplement: Figure S4 — Distribution of partial correlation coefficients. Partial correlations center around zero with a shift towards positive high values. When applying a correlation cutoff of r = 0.3, we are left with 109 out of 8515 correlation values (1.28%). (TIFF) [file pgen.1002215.s004.tif]

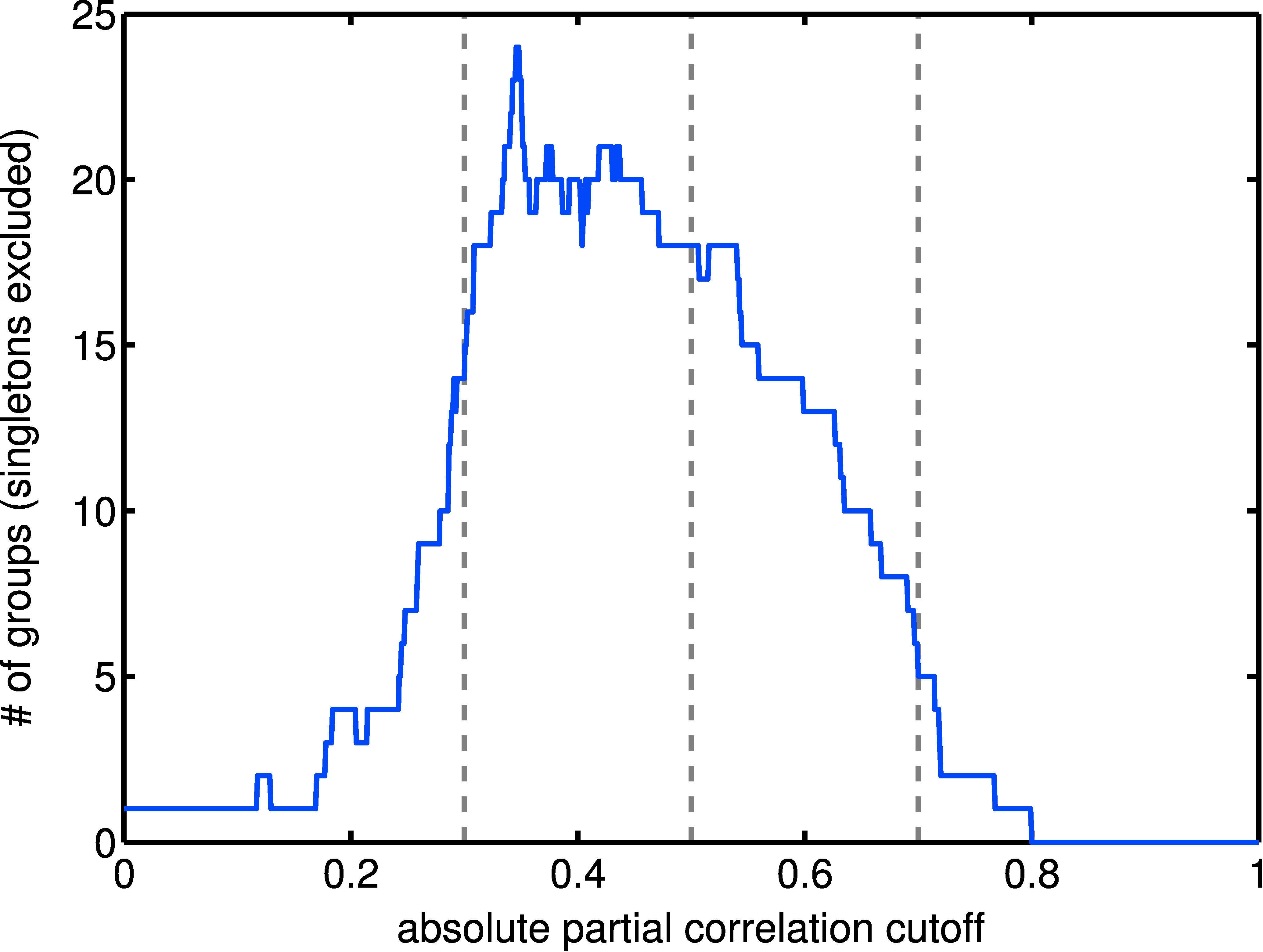

Supplement: Figure S5 — Number of clustered groups in the GGM as a function of the absolute partial correlation cutoff. Note that we did not count singleton metabolites that is metabolites without any partial correlation above threshold, here. Most non-singleton groups emerge in the cutoff range between 0.3 and 0.7, which corresponds to the figure in the main manuscript. For our lower cutoff of 0.3, we obtain 14 groups, which can here be regarded as independent phenotypes in the metabolite pool. (TIFF) [file pgen.1002215.s005.tif]
