# Supplementary material for: Discovery of Sexual Dimorphisms in Metabolic and Genetic Biomarkers
Source: PLoS Genet. 2011 Aug 11;7(8):e1002215. doi: 10.1371/journal.pgen.1002215 (PMC3154959; doi:10.1371/journal.pgen.1002215)
Supplement: Table S1 — Study population characteristics. Data are presented as mean (SD) or number of persons (N); BMI indicates body mass index; HDL high density lipoprotein; LDL low density lipoprotein; smokers: number of smokers with one or more than one cigarette/day, high alcohol intake: subjects were counted for high alcohol intake when they had an alcohol consumption of ≥0 g alcohol/day for males and ≥20 g alcohol/day for females. (A) Study populations used for phenotypic analysis. (B) Study populations used for genotypic analysis. (DOCX) [file pgen.1002215.s006.docx]

**Table S1**

**A**

**B**
