# Supplementary material for: Discovery of Sexual Dimorphisms in Metabolic and Genetic Biomarkers
Source: PLoS Genet. 2011 Aug 11;7(8):e1002215. doi: 10.1371/journal.pgen.1002215 (PMC3154959; doi:10.1371/journal.pgen.1002215)
Supplement: Table S3 — Phenotypic metabotype differences between males and females of the replication sample KORA F3. P-values were calculated by a linear regression model with metabolite concentration as outcome and sex as explanatory variable adjusted for age, BMI and waist-hip ratio (WHR). Gray shaded columns show significant p-values for differences in the metabolite concentrations between males and females after Bonferroni correction (significance level after multiple testing = p-value<3.8×10^−4^). (DOCX) [file pgen.1002215.s008.docx]

ß=beta-estimate of linear regression model; r^2^=explained variance; BMI=Body Mass Index;
