# Supplementary material for: Discovery of Sexual Dimorphisms in Metabolic and Genetic Biomarkers
Source: PLoS Genet. 2011 Aug 11;7(8):e1002215. doi: 10.1371/journal.pgen.1002215 (PMC3154959; doi:10.1371/journal.pgen.1002215)
Supplement: Table S4 — Comparison of different adjustments in association of SNPs with glycine. Results for SNPs which showed a significant difference in beta-estimates for KORA F4 with the adjustment of sex-specific GWAs for BMI (age, batch), for different adjustment for waist-hip ratio (WHR) (age, batch) or adjustment for WHR and BMI (age, batch). (DOCX) [file pgen.1002215.s009.docx]

**Table S4. Comparison of different adjustments in association of SNPs with glycine.**
