# Supplementary material for: Discovery of Sexual Dimorphisms in Metabolic and Genetic Biomarkers
Source: PLoS Genet. 2011 Aug 11;7(8):e1002215. doi: 10.1371/journal.pgen.1002215 (PMC3154959; doi:10.1371/journal.pgen.1002215)
Supplement: Table S5 — Detailed information for SNPs with significant gender differences in beta-estimates for association with glycine. Minor allele frequency is calculated for men and women separately in each study. Imputation quality (RSQ) respectively call rate for genotyped SNPs is calculated based on all IDs. (DOCX) [file pgen.1002215.s010.docx]

**Table S5. Detailed information for SNPs with significant gender differences in beta-estimates for association with glycine**. Minor allele frequency is calculated for men and women separately in each study. Imputation quality (RSQ) respectively call rate for genotyped SNPs is calculated based on all IDs.
