# Supplementary material for: Discovery of Sexual Dimorphisms in Metabolic and Genetic Biomarkers
Source: PLoS Genet. 2011 Aug 11;7(8):e1002215. doi: 10.1371/journal.pgen.1002215 (PMC3154959; doi:10.1371/journal.pgen.1002215)
Supplement: Text S1 — Metabolite panel. (DOC) [file pgen.1002215.s014.doc]

*Text S1 to*

**Discovery of sexual dimorphisms in metabolic and genetic biomarkers**

Kirstin Mittelstrass *et al*.

**CONTENTS**

**TEXT S1**

**FIGURES**

| Figure S1: | KORA study populations |
| --- | --- |
| Figure S2: | QQ-plots for the sex-stratified GWAs with metabolic trait glycine |
| Figure S3: | Regional association plots for sex-stratified GWAS with glycine around the locus *CPS1* |
| Figure S4: | Distribution of partial correlation coefficients. |
| Figure S5: | Number of clustered groups in the GGM as a function of the absolute partial correlation cutoff. |

TABLES

| Table S1: | Study population characteristic |
| --- | --- |
| Table S2: | Phenotypic metabotype differences between males and females of the discovery sample KORA F4 |
| Table S3: | Phenotypic metabotype differences between males and females of the replication sample KORA F3 |
| Table S4: | Comparison of different adjustments in association of SNPs with glycine. |
| Table S5: | Detailed information for SNPs with significant gender differences in beta-estimates for association with glycine |
| Table S6: | Full biochemical names of all 131 metabolites used for further analysis that were measured on the Biocrates AbsoluteIDQ kit |
| Table S7: | Excluded metabolites that were measured on the Biocrates AbsoluteIDQ kit. |
| Table S8: | Metabolite concentrations of the study cohorts KORA F4 and KORA F3 |

**Metabolite panel:** The metabolomics dataset contains amino acids, free carnitine (C0), acylcarnitines (C*x:y*), hydroxylacylcarnitines (C(OH)*x:y*), different sphingomyelins (SM*x:y*) and sphingomyelin-derivatives, such as N- hydroxylacyloylsphingosylphosphocholine (SM (OH)*x:y*). In addition, various phosphatidylcholines (PC) were quantified. Phosphatidylcholines are differentiated with respect to the presence of ester (*a*) and ether (*e*) bonds in the glycerol moiety, where two letters (*aa*=diacyl, *ae*=acyl-alkyl) denote that two glycerol positions are bound to a fatty acid residue, while a single letter (*a*=acyl) indicates the presence of a single fatty acid residue. Lipid side chain composition is abbreviated as C*x:y*, where *x* denotes the number of carbons in the side chain and *y* the number of double bonds. The precise position of the double bonds and the distribution of the carbon atoms in different fatty acid side chains cannot be determined with this technology. In some cases, the mapping of metabolite names to individual masses can be ambiguous. For example, stereo-chemical differences are not always discernible, neither are isobaric fragments. In such cases, possible alternative assignments are indicated (**Table S6**).
