# Supplementary material for: Discovery of Sexual Dimorphisms in Metabolic and Genetic Biomarkers
Source: PLoS Genet. 2011 Aug 11;7(8):e1002215. doi: 10.1371/journal.pgen.1002215 (PMC3154959; doi:10.1371/journal.pgen.1002215)
Supplement: Supplementary file 8 [file pgen.1002215.s011.docx]

Abbreviation	Biochemical name	r	Above LOD (%)	CV (%)
C0	Carnitine	0.88	100.0	6.7
C2	Acetylcarnitine	0.94	100.0	9.4
C3	Propionylcarnitine	0.86	100.0	8.0
C4	Butyrylcarnitine	0.89	100.0	8.8
C4:1	Hexanoylcarnitine (Fumarylcarnitine)	0.85	76.7	13.6
C5	Valerylcarnitine	0.81	95.6	14.2
C8	Octanoylcarnitine	0.89	51.5	16.3
C8:1	Octenoylcarnitine	0.92	96.0	8.4
C9	Nonaylcarnitine	0.84	83.7	20.8
C10	Decanoylcarnitine	0.93	94.1	11.4
C10:1	Decenoylcarnitine	0.83	48.7	10.4
C10:2	Decadienylcarnitine	0.51	50.5	14.5
C12	Dodecanoylcarnitine	0.86	87.4	10.4
C12:1	Dodecenoylcarnitine	0.73	13.7	13.0
C14	Tetradecanoylcarnitine	0.54	51.7	12.6
C14:1	Tetradecenoylcarnitine	0.81	100.0	16.9
C14:1, OH	Hydroxytetradecenoylcarnitine	0.70	67.4	16.4
C14:2	Tetradecadienylcarnitine	0.87	98.8	11.6
C16	Hexadecanoylcarnitine	0.84	100.0	8.9
C16:1	Hexadecenoylcarnitine	0.71	2.8	10.2
C16:2	Hexadecadienylcarnitine	0.57	70.7	19.4
C18	Octadecanoylcarnitine	0.69	99.8	13.7
C18:1	Octadecenoylcarnitine	0.87	98.3	10.2
C18:2	Octadecadienylcarnitine	0.81	100.0	9.4
Arg	Arginine	0.59	100.0	8.2
Gln	Glutamine	0.62	100.0	9.9
Gly	Glycine	0.89	100.0	7.9
His	Histidine	0.69	100.0	8.3
Met	Methionine	0.53	100.0	9.7
Orn	Ornithine	0.75	100.0	9.4
Phe	Phenylalanine	0.62	100.0	8.4
Pro	Proline	0.89	100.0	7.4
Ser	Serine	0.62	100.0	9.6

Abbreviation	Biochemical name	r	Above LOD (%)	CV (%)
**Thr**	**Threonine**	**0.71**	**100.0**	**12.1**
Trp	Tryptophan	0.51	100.0	7.5
**Tyr**	**Tyrosine**	**0.66**	**100.0**	**8.6**
Val	Valine	0.69	100.0	19.6
**xLeu**	**Leucine/Isoleucine**	**0.74**	**100.0**	**8.2**
PC aa C28:1	Phosphatidylcholine diacyl C28:1	0.87	100.0	9.8
**PC aa C30:0**	**Phosphatidylcholine diacyl C30:0**	**0.89**	**100.0**	**7.8**
PC aa C32:0	Phosphatidylcholine diacyl C32:0	0.83	100.0	7.1
**PC aa C32:1**	**Phosphatidylcholine diacyl C32:1**	**0.96**	**100.0**	**7.4**
PC aa C32:2	Phosphatidylcholine diacyl C32:2	0.91	99.9	11.1
**PC aa C32:3**	**Phosphatidylcholine diacyl C32:3**	**0.79**	**100.0**	**8.9**
PC aa C34:1	Phosphatidylcholine diacyl C34:1	0.83	100.0	7.2
**PC aa C34:2**	**Phosphatidylcholine diacyl C34:2**	**0.75**	**100.0**	**7.7**
PC aa C34:3	Phosphatidylcholine diacyl C34:3	0.91	100.0	8.6
**PC aa C34:4**	**Phosphatidylcholine diacyl C34:4**	**0.92**	**100.0**	**8.0**
PC aa C36:0	Phosphatidylcholine diacyl C36:0	0.74	100.0	17.4
**PC aa C36:1**	**Phosphatidylcholine diacyl C36:1**	**0.84**	**100.0**	**8.5**
PC aa C36:2	Phosphatidylcholine diacyl C36:2	0.80	100.0	6.7
**PC aa C36:3**	**Phosphatidylcholine diacyl C36:3**	**0.86**	**100.0**	**7.5**
PC aa C36:4	Phosphatidylcholine diacyl C36:4	0.87	100.0	7.8
**PC aa C36:5**	**Phosphatidylcholine diacyl C36:5**	**0.82**	**100.0**	**8.6**
PC aa C36:6	Phosphatidylcholine diacyl C36:6	0.89	100.0	11.1
**PC aa C38:0**	**Phosphatidylcholine diacyl C38:0**	**0.86**	**100.0**	**13.8**
PC aa C38:3	Phosphatidylcholine diacyl C38:3	0.86	100.0	7.6
**PC aa C38:4**	**Phosphatidylcholine diacyl C38:4**	**0.88**	**100.0**	**7.3**
PC aa C38:5	Phosphatidylcholine diacyl C38:5	0.83	100.0	7.9
**PC aa C38:6**	**Phosphatidylcholine diacyl C38:6**	**0.93**	**100.0**	**8.1**
PC aa C40:1	Phosphatidylcholine diacyl C40:1	0.51	8.7	13.5
**PC aa C40:2**	**Phosphatidylcholine diacyl C40:2**	**0.51**	**100.0**	**11.7**
PC aa C40:3	Phosphatidylcholine diacyl C40:3	0.60	100.0	11.2
**PC aa C40:4**	**Phosphatidylcholine diacyl C40:4**	**0.86**	**100.0**	**7.6**
PC aa C40:5	Phosphatidylcholine diacyl C40:5	0.89	100.0	7.0
**PC aa C40:6**	**Phosphatidylcholine diacyl C40:6**	**0.93**	**100.0**	**7.1**
PC aa C42:0	Phosphatidylcholine diacyl C42:0	0.85	100.0	12.3
**PC aa C42:1**	**Phosphatidylcholine diacyl C42:1**	**0.72**	**100.0**	**14.8**
PC aa C42:2	Phosphatidylcholine diacyl C42:2	0.56	100.0	14.6
**PC aa C42:4**	**Phosphatidylcholine diacyl C42:4**	**0.51**	**100.0**	**11.7**
PC aa C42:5	Phosphatidylcholine diacyl C42:5	0.75	100.0	10.6
**PC aa C42:6**	**Phosphatidylcholine diacyl C42:6**	**0.62**	**60.2**	**12.5**
PC ae C30:0	Phosphatidylcholine acyl-alkyl C30:0	0.76	98.9	18.1
**PC ae C30:2**	**Phosphatidylcholine acyl-alkyl C30:2**	**0.65**	**86.3**	**17.5**
PC ae C32:1	Phosphatidylcholine acyl-alkyl C32:1	0.83	100.0	8.0
**PC ae C32:2**	**Phosphatidylcholine acyl-alkyl C32:2**	**0.77**	**100.0**	**11.6**

Abbreviation	Biochemical name	r	Above LOD (%)	CV (%)
PC ae C34:0	Phosphatidylcholine acyl-alkyl C34:0	0.82	100.0	7.9
PC ae C34:1	Phosphatidylcholine acyl-alkyl C34:1	0.87	100.0	7.6
PC ae C34:2	Phosphatidylcholine acyl-alkyl C34:2	0.90	100.0	7.6
PC ae C34:3	Phosphatidylcholine acyl-alkyl C34:3	0.91	100.0	7.9
PC ae C36:1	Phosphatidylcholine acyl-alkyl C36:1	0.85	100.0	9.8
PC ae C36:2	Phosphatidylcholine acyl-alkyl C36:2	0.92	100.0	8.3
PC ae C36:3	Phosphatidylcholine acyl-alkyl C36:3	0.86	100.0	8.1
PC ae C36:4	Phosphatidylcholine acyl-alkyl C36:4	0.87	100.0	7.9
PC ae C36:5	Phosphatidylcholine acyl-alkyl C36:5	0.89	100.0	8.0
PC ae C38:0	Phosphatidylcholine acyl-alkyl C38:0	0.81	100.0	10.8
PC ae C38:1	Phosphatidylcholine acyl-alkyl C38:1	0.48	100.0	12.4
PC ae C38:2	Phosphatidylcholine acyl-alkyl C38:2	0.73	100.0	10.3
PC ae C38:3	Phosphatidylcholine acyl-alkyl C38:3	0.85	100.0	9.2
PC ae C38:4	Phosphatidylcholine acyl-alkyl C38:4	0.82	100.0	8.6
PC ae C38:5	Phosphatidylcholine acyl-alkyl C38:5	0.82	100.0	8.3
PC ae C38:6	Phosphatidylcholine acyl-alkyl C38:6	0.85	100.0	8.1
PC ae C40:0	Phosphatidylcholine acyl-alkyl C40:0	0.87	1.0	4.8
PC ae C40:1	Phosphatidylcholine acyl-alkyl C40:1	0.68	100.0	10.5
PC ae C40:2	Phosphatidylcholine acyl-alkyl C40:2	0.85	100.0	9.5
PC ae C40:3	Phosphatidylcholine acyl-alkyl C40:3	0.73	100.0	9.5
PC ae C40:4	Phosphatidylcholine acyl-alkyl C40:4	0.82	100.0	9.6
PC ae C40:5	Phosphatidylcholine acyl-alkyl C40:5	0.78	100.0	8.3
PC ae C40:6	Phosphatidylcholine acyl-alkyl C40:6	0.88	100.0	8.6
PC ae C42:0	Phosphatidylcholine acyl-alkyl C42:0	0.60	14.9	15.7
PC ae C42:1	Phosphatidylcholine acyl-alkyl C42:1	0.51	100.0	11.5
PC ae C42:2	Phosphatidylcholine acyl-alkyl C42:2	0.69	100.0	12.8
PC ae C42:3	Phosphatidylcholine acyl-alkyl C42:3	0.80	100.0	10.8
PC ae C42:4	Phosphatidylcholine acyl-alkyl C42:4	0.78	100.0	9.2
PC ae C42:5	Phosphatidylcholine acyl-alkyl C42:5	0.86	100.0	7.4
PC ae C44:3	Phosphatidylcholine acyl-alkyl C44:3	0.50	100.0	12.5
PC ae C44:4	Phosphatidylcholine acyl-alkyl C44:4	0.71	100.0	11.4
PC ae C44:5	Phosphatidylcholine acyl-alkyl C44:5	0.86	100.0	8.0
PC ae C44:6	Phosphatidylcholine acyl-alkyl C44:6	0.89	100.0	7.7
lysoPC a C16:0	lysoPhosphatidylcholine acyl C16:0	0.75	100.0	8.8
lysoPC a C16:1	lysoPhosphatidylcholine acyl C16:1	0.84	100.0	8.6
lysoPC a C17:0	lysoPhosphatidylcholine acyl C17:0	0.84	100.0	12.7
lysoPC a C18:0	lysoPhosphatidylcholine acyl C18:0	0.80	100.0	9.7
lysoPC a C18:1	lysoPhosphatidylcholine acyl C18:1	0.84	100.0	9.2
lysoPC a C18:2	lysoPhosphatidylcholine acyl C18:2	0.93	100.0	8.8
lysoPC a C20:3	lysoPhosphatidylcholine acyl C20:3	0.77	100.0	9.0
lysoPC a C20:4	lysoPhosphatidylcholine acyl C20:4	0.87	100.0	9.0

Abbreviation	Biochemical name	r	Above LOD (%)	CV (%)
SM (OH) C14:1	Hydroxysphingomyeline C14:1	0.91	100.0	7.7
SM (OH) C16:1	Hydroxysphingomyeline C16:1	0.86	100.0	8.8
SM (OH) C22:1	Hydroxysphingomyeline C22:1	0.82	100.0	11.2
SM (OH) C22:2	Hydroxysphingomyeline C22:2	0.87	100.0	10.3
SM (OH) C24:1	Hydroxysphingomyeline C24:1	0.75	100.0	15.1
SM C16:0	Sphingomyeline C16:0	0.73	100.0	8.0
SM C16:1	Sphingomyeline C16:1	0.84	100.0	7.5
SM C18:0	Sphingomyeline C18:0	0.79	100.0	9.0
SM C18:1	Sphingomyeline C18:1	0.84	100.0	8.2
SM C20:2	Sphingomyeline C20:2	0.61	99.9	12.6
SM C24:0	Sphingomyeline C24:0	0.78	100.0	10.7
SM C24:1	Sphingomyeline C24:1	0.75	100.0	10.0
SM C26:1	Sphingomyeline C26:1	0.69	100.0	20.8
H1	Hexose	0.69	100.0	6.3
