# Supplementary material for: Discovery of Sexual Dimorphisms in Metabolic and Genetic Biomarkers
Source: PLoS Genet. 2011 Aug 11;7(8):e1002215. doi: 10.1371/journal.pgen.1002215 (PMC3154959; doi:10.1371/journal.pgen.1002215)
Supplement: Supplementary file 9 [file pgen.1002215.s012.docx]

Abbreviation	Biochemical name	r	Above LOD (%)	CV (%)
C3.OH	Hydroxypropionylcarnitine	0.05	0.4	0.8
C3:1	Propenoylcarnitine	-0.11	0.1	0.4
C4-OH(C3-DC)	Hydroxybutyrylcarnitine	0.47	8.4	0.4
C4:1	Butenylcarnitine	0.04	5.7	0.3
C5-DC(C6-OH)	Glutarylcarnitine (Hydroxyhexanoylcarnitine)	0.15	27.1	0.2
C5.M.DC	Methylglutarylcarnitine	0.18	0.9	0.4
C5-OH (C3-DC-M)	Hydroxyvalerylcarnitine (Methylmalonylcarnitine)	0.25	55.1	0.3
C5:1	Tiglylcarnitine	0.37	0.8	0.3
C5:1.DC	Glutaconylcarnitine	0.13	12.5	0.4
C6:1	Hexenoylcarnitine	0.07	0.3	0.3
C7.DC	Pimelylcarnitine	0.79	61.3	0.3
C12.DC	Dodecanedioylcarnitine	0.05	0.0	0.1
C14:2.OH	Hydroxytetradecadienylcarnitine	0.27	38.0	0.2
C16.OH	Hydroxyhexadecanoylcarnitine	0.20	3.3	0.2
C16:1.OH	Hydroxyhexadecenoylcarnitine	0.38	2.3	0.2
C16:2.OH	Hydroxyhexadecadienylcarnitine	0.32	4.7	0.2
C18:1.OH	Hydroxyoctadecenoylcarnitine	0.06	0.9	0.3
PC aa C24:0	Phosphatidylcholine diacyl C24:0	0.11	72.5	0.3
PC aa C26:0	Phosphatidylcholine diacyl C26:0	0.09	11.5	0.3
PC aa C30:2	Phosphatidylcholine diacyl C30:2	0.12	4.2	0.8
PC aa C38:1	Phosphatidylcholine diacyl C38:1	0.34	99.8	0.2
PC ae C30:1	Phosphatidylcholine acyl-alkyl C30:1	0.18	94.1	0.4
PC ae C36:0	Phosphatidylcholine acyl-alkyl C36:0	0.35	100.0	0.4
lysoPC a C14:0	lysoPhosphatidylcholine acyl C14:0	0.45	21.2	0.2
lysoPC a C24:0	lysoPhosphatidylcholine acyl C24:0	0.09	12.5	0.2
lysoPC a C26:0	lysoPhosphatidylcholine acyl C26:0	0.09	59.6	0.3
lysoPC a C26:1	lysoPhosphatidylcholine acyl C26:1	-0.04	0.0	0.1
lysoPC a C28:0	lysoPhosphatidylcholine acyl C28:0	0.17	49.6	0.3
lysoPC a C28:1	lysoPhosphatidylcholine acyl C28:1	0.29	99.8	0.2
lysoPC a C6:0	lysoPhosphatidylcholine acyl C6:0	-0.14	33.3	0.6
SM C22:3	Sphingomyeline C22:3	-0.04	55.8	0.6
SM C26:0	Sphingomyeline C26:0	0.46	100.0	0.7
