# Supplementary material for: Discovery of Sexual Dimorphisms in Metabolic and Genetic Biomarkers
Source: PLoS Genet. 2011 Aug 11;7(8):e1002215. doi: 10.1371/journal.pgen.1002215 (PMC3154959; doi:10.1371/journal.pgen.1002215)
Supplement: Supplementary file 10 [file pgen.1002215.s013.docx]

	KORA F4			KORA F3		
metabolites	male (µmol/l)	female (µmol/l)	Δ (%)	male (µmol/l)	female (µmol/l)	Δ (%)
**acylcarnitines**						
C0	38.25 ± 7.71	33.51 ± 7.22	14.1%	41.9 ± 9.4	40.7 ± 9.01	2.9%
C10	0.38 ± 0.18	0.34 ± 0.15	10.9%	0.51 ± 0.56	0.44 ± 0.16	16.6%
C10:1	0.18 ± 0.07	0.16 ± 0.06	10.6%	0.23 ± 0.12	0.20 ± 0.06	10.6%
C10:2	0.044 ± 0.01	0.039 ± 0.01	10.5%	0.057± 0.02	0.056 ± 0.02	1.2%
C12	0.14 ± 0.06	0.12 ± 0.05	13.3%	0.15 ± 0.08	0.13 ± 0.04	12.2%
C12:1	0.15 ± 0.06	0.14 ± 0.05	9.3%	0.17 ± 0.06	0.16 ± 0.05	4.5%
C14	0.05 ± 0.01	0.04 ± 0.01	6.9%	0.061 ± 0.03	0.058 ± 0.02	5.0%
C14:1	0.151 ± 0.05	0.145 ± 0.04	3.7%	0.152 ± 0.07	0.145 ± 0.04	4.7%
C14:1-OH	0.02 ± 0.005	0.01 ± 0.004	10.7%	0.041 ± 0.02	0.036 ± 0.01	4.3%
C14:2	0.034 ± 0.02	0.029 ± 0.01	14.4%	0.014 ± 0.005	0.013 ± 0.004	14.2%
C16	0.13 ± 0.03	0.11 ± 0.03	13.3%	0.14 ± 0.04	0.13 ± 0.03	7.5%
C16:1	0.038 ± 0.01	0.037 ± 0.01	1.3%	0.054 ± 0.02	0.053 ± 0.01	0.5%
C16:2	0.008 ± 0.003	0.007 ± 0.003	8.4%	0.011 ± 0.01	0.010 ± 0.004	5.7%
C18	0.055 ± 0.01	0.047 ± 0.01	15.1%	0.064 ± 0.02	0.058 ± 0.01	9.0%
C18:1	0.14 ± 0.04	0.13 ± 0.03	10.2%	0.15 ± 0.04	0.14 ± 0.04	5.4%
C18:2	0.05 ± 0.02	0.04 ± 0.01	16.5%	0.06 ± 0.02	0.05 ± 0.01	12.9%
C2	8.37 ± 2.99	8.12 ± 2.7	3.1%	9.56 ± 3.82	9.28 ± 2.89	3.0%
C3	0.44 ± 0.14	0.36 ± 0.11	22.4%	0.97 ± 0.32	0.93 ± 0.31	4.7%
C4	0.24 ± 0.12	0.22 ± 0.1	11.4%	0.340 ± 0.25	0.343 ± 0.16	-0.9%
C4:1	0.08 ± 0.03	0.07 ± 0.02	8.3%	0.028 ± 0.01	0.029 ± 0.01	-3.1%
C5	0.13 ± 0.04	0.11 ± 0.03	27.8%	0.154 ± 0.05	0.146 ± 0.06	5.3%
C8	0.23 ± 0.1	0.21 ± 0.09	9.2%	0.28 ± 0.35	0.24 ± 0.09	17.3%
C8:1	0.094 ± 0.05	0.088 ± 0.05	7.1%	0.11 ± 0.05	0.12 ± 0.06	-9.8%
C9	0.054 ± 0.03	0.049 ± 0.02	7.9%	0.074 ± 0.04	0.072 ± 0.03	2.1%
**amino acids**						
Arg	116.6 ± 19.5	115.3 ± 19.4	1.1%	96.8 ± 15.9	94.6 ± 18.1	2.3%
Gln	627.5 ± 95.8	604.4 ± 92.1	3.8%	525.2 ± 76.9	513.2 ± 79.5	2.3%
Gly	284.4 ± 59.5	330.8 ± 94.8	-14.0%	251.3 ± 62.8	283.2 ± 83.8	-11.3%
His	99.6 ± 14.4	96.6 ± 13.9	3.1%	93.3 ± 18.9	91.1 ± 20.2	2.4%
Met	33.3 ± 5.8	30.1 ± 5.1	10.7%	31.2 ± 5.7	28.7 ± 5.2	8.7%
Orn	84.4 ± 18.8	78.5 ± 19	7.5%	102.8 ± 22.2	104.8 ± 27.1	-2.0%
Phe	64.1 ± 10.6	60.1 ± 9.7	6.7%	76.4 ± 22.3	72.5 ± 20.2	5.4%
Pro	192.2 ± 52.1	159.2 ± 51.2	20.7%	208.2 ± 53.4	186.6 ± 57.5	11.6%
Ser	124.6 ± 22.4	132.1 ± 26.2	-5.7%	78.3 ± 19.4	83.2 ± 22.4	-5.9%
Thr	105.7 ± 22.4	105.4 ± 26.4	0.3%	99.6 ± 22.2	98.4 ± 21.2	1.2%
Trp	85.1 ± 10.5	80.2 ± 9.2	6.1%	80.5 ± 10.2	77 ± 12.1	4.6%
Tyr	88.6 ± 17.6	81.8 ± 17.5	8.3%	84.7 ± 18.97	83.3 ± 20.18	1.7%
Val	295.4 ± 53.2	254 ± 47.7	16.3%	265.8 ± 47.7	243.3 ± 52.2	9.3%
xLeu	237.1 ± 43.4	191.6 ± 35	23.8%	234.9 ± 46.4	201.6 ± 48.6	16.5%

	KORA F4			KORA F3		
metabolites	male (µmol/l)	female (µmol/l)	Δ (%)	male (µmol/l)	female (µmol/l)	Δ (%)
**phosphatidylcholines**						
PC aa C28:1	3.1 ± 0.7	3.6 ± 0.9	-12.8%	3.3 ± 0.8	4.01 ± 0.95	-17.0%
PC aa C30:0	4.4 ± 1.4	5 ± 1.5	-10.4%	6.2 ± 1.9	6.99 ± 1.9	-11.1%
PC aa C32:0	14.9 ± 3.1	15.3 ± 3	-2.5%	19.2 ± 4.8	20.1 ± 4.6	-4.7%
PC aa C32:1	21.1 ± 12	22.3 ± 10.3	-5.1%	32.8 ± 15.5	35.1 ± 11.1	-6.5%
PC aa C32:2	3.5 ± 1.4	4.2 ± 1.6	-16.8%	8.6 ± 2.2	10.5 ± 2.3	-17.7%
PC aa C32:3	0.4 ± 0.09	0.5 ± 0.12	-17.9%	0.50 ± 0.12	0.63 ± 0.16	-20.9%
PC aa C34:1	238.6 ± 56.4	241 ± 53.3	-1.0%	290.1 ± 73.1	299.7 ± 68.4	-3.2%
PC aa C34:2	380.9 ± 66.7	396.6 ± 64.6	-4.0%	391.4 ± 102.1	414.5 ± 103.7	-5.6%
PC aa C34:3	16.6 ± 4.8	19.2 ± 5.2	-13.6%	18.7 ± 5.9	22.4 ± 6.3	-16.5%
PC aa C34:4	2.1 ± 0.8	2.4 ± 0.9	-11.6%	2.4 ± 1	2.8 ± 0.96	-15.5%
PC aa C36:0	2.6 ± 0.8	2.8 ± 0.8	-4.5%	8.6 ± 1.9	9.7 ± 2.3	-10.8%
PC aa C36:1	52.8 ± 13.8	54.5 ± 12.7	-3.1%	81.0 ± 18.7	89.3 ± 20.4	-9.2%
PC aa C36:2	225.6 ± 44.2	238.3 ± 41.9	-5.3%	247.6 ± 51.8	274.7 ± 55.2	-9.9%
PC aa C36:3	145.6 ± 30.7	154.1 ± 31.3	-5.5%	179.1 ± 44.2	193.2 ± 40.1	-7.3%
PC aa C36:4	218.6 ± 52.4	221.4 ± 51.1	-1.3%	230 ± 58.3	240.4 ± 51.7	-4.3%
PC aa C36:5	29.5 ± 14.6	29.1 ± 13.3	1.3%	32.5 ± 20.3	36.9 ± 17.2	-12.0%
PC aa C36:6	1.0 ± 0.4	1.2 ± 0.5	-12.8%	1.4 ± 0.6	1.6 ± 0.6	-15.5%
PC aa C38:0	3.2 ± 0.9	3.4 ± 0.9	-7.2%	3.9 ± 1.03	4.6 ± 1.4	-14.4%
PC aa C38:3	52.3 ± 13.4	55.5 ± 13.3	-5.8%	67.1 ± 17.7	76.6 ± 18.3	-12.5%
PC aa C38:4	118.2 ± 32.2	121 ± 29.5	-2.3%	125.4 ± 32.5	138.6 ± 32.5	-9.5%
PC aa C38:5	61.5 ± 15.6	62.9 ± 14.4	-2.3%	74.3 ± 21	82.1 ± 21.2	-9.5%
PC aa C38:6	88.4 ± 26.3	92.5 ± 27.3	-4.4%	92.7 ± 28.5	100.7 ± 31.4	-8.0%
PC aa C40:1	0.46 ± 0.09	0.48 ± 0.1	-4.7%	0.7 ± 0.1	0.7 ± 0.2	-7.1%
PC aa C40:2	0.355 ± 0.1	0.362 ± 0.1	-1.8%	0.6 ± 0.2	0.7 ± 0.2	-6.4%
PC aa C40:3	0.6 ± 0.2	0.7 ± 0.2	-2.5%	1.1 ± 0.3	1.2 ± 0.3	-5.3%
PC aa C40:4	4.2 ± 1.3	4.1 ± 1.1	1.4%	5.5 ± 1.7	5.7 ± 1.5	-3.7%
PC aa C40:5	11.53 ± 3.4	11.43 ± 3	0.6%	15.5 ± 4.6	16.8 ± 4.5	-8.1%
PC aa C40:6	28.2 ± 9.6	29.2 ± 9.5	-3.4%	30.6 ± 9.9	34.8 ± 11.8	-12.0%
PC aa C42:0	0.56 ± 0.2	0.64 ± 0.2	-12.9%	0.6 ± 0.2	0.7 ± 0.2	-11.8%
PC aa C42:1	0.3 ± 0.08	0.3 ± 0.09	-10.2%	0.3 ± 0.1	0.3 ± 0.1	-8.7%
PC aa C42:2	0.21 ± 0.06	0.22 ± 0.06	-3.2%	0.2 ± 0.1	0.2 ± 0.1	-3.7%
PC aa C42:4	0.215 ± 0.05	0.224 ± 0.05	-3.7%	0.3 ± 0.1	0.3 ± 0.1	-4.2%
PC aa C42:5	0.42 ± 0.1	0.44 ± 0.1	-4.0%	0.5 ± 0.2	0.6 ± 0.2	-6.6%
PC aa C42:6	0.61 ± 0.1	0.64 ± 0.1	-4.3%	0.7 ± 0.2	0.8 ± 0.2	-6.8%
**phosphatidylcholines**						
PC ae C30:0	0.4 ± 0.1	0.5 ± 0.1	-13.9%	0.5 ± 0.1	0.6 ± 0.2	-14.6%
PC ae C30:2	0.1 ± 0.04	0.2 ± 0.04	-14.2%	0.2 ± 0.04	0.2 ± 0.05	-17.1%
PC ae C32:1	2.7 ± 0.6	3.0 ± 0.6	-10.6%	4.4 ± 0.9	5.01 ± 1.2	-12.7%
PC ae C32:2	0.7 ± 0.1	0.8 ± 0.2	-16.3%	0.7 ± 0.2	0.9 ± 0.2	-17.9%
PC ae C34:0	1.6 ± 0.4	1.8 ± 0.5	-10.8%	2.97 ± 0.7	3.4 ± 0.8	-12.5%
PC ae C34:1	9.8 ± 2	11.2 ± 2.3	-13.1%	12.2 ± 2.6	14.1 ± 3.2	-13.1%

	KORA F4			KORA F3		
metabolites	male (µmol/l)	female (µmol/l)	Δ (%)	male (µmol/l)	female (µmol/l)	Δ (%)
PC ae C34:2	11.7 ± 3	13.5 ± 3.2	-13.1%	13.4 ± 3.5	15.8 ± 4.4	-15.5%
PC ae C34:3	7.8 ± 2.3	9.0 ± 2.5	-13.1%	8.2 ± 2.5	9.5 ± 3.1	-14.1%
PC ae C36:1	7.8 ± 1.72	8.9 ± 2.1	-12.3%	10.4 ± 2.7	11.9 ± 3	-13.0%
PC ae C36:2	13.8 ± 3.4	16.4 ± 3.8	-15.8%	16.6 ± 4.01	19.6 ± 5.07	-15.4%
PC ae C36:3	8.1 ± 1.9	9.0 ± 2	-10.3%	11.1 ± 2.5	12.6 ± 3.1	-12.1%
PC ae C36:4	21.0 ± 5.6	20.7 ± 5	1.7%	22.8 ± 5.9	24.6 ± 6.3	-7.4%
PC ae C36:5	13.9 ± 3.6	13.8 ± 3.5	0.5%	14.3 ± 3.7	15.6 ± 4.2	-8.7%
PC ae C38:0	2.3 ± 0.7	2.6 ± 0.8	-10.5%	2.6 ± 0.9	3.1 ± 1.1	-16.9%
PC ae C38:1	0.8 ± 0.3	0.8 ± 0.3	-6.3%	6.4 ± 1.4	7.6 ± 1.7	-15.8%
PC ae C38:2	2.0 ± 0.4	2.3 ± 0.5	-11.9%	6.98 ± 1.5	8.6 ± 2.0	-18.8%
PC ae C38:3	4.0 ± 0.9	4.6 ± 1	-13.9%	6.1 ± 1.4	7.0 ± 1.6	-13.0%
PC ae C38:4	15.2 ± 3.1	16.2 ± 3.2	-6.4%	18 ± 3.8	19.6 ± 4.2	-8.3%
PC ae C38:5	19.9 ± 4.4	19.9 ± 4.2	0.1%	21.9 ± 4.6	23.7 ± 5.2	-7.3%
PC ae C38:6	8.5 ± 2.1	8.9 ± 2.2	-5.1%	8.7 ± 2.2	10.2 ± 3	-14.8%
PC ae C40:0	10.0 ± 1.5	10.5 ± 1.6	-4.8%	10.7 ± 2.3	11.4 ± 2.7	-6.6%
PC ae C40:1	1.65 ± 0.4	1.67 ± 0.4	-2.1%	2.4 ± 0.5	2.7 ± 0.7	-9.6%
PC ae C40:2	2.0 ± 0.5	2.2 ± 0.5	-10.6%	2.2 ± 0.5	2.5 ± 0.6	-14.1%
PC ae C40:3	1.0 ± 0.2	1.2 ± 0.2	-15.1%	1.4 ± 0.3	1.6 ± 0.4	-12.8%
PC ae C40:4	2.5 ± 0.49	2.7 ± 0.5	-8.7%	2.9 ± 0.6	3.2 ± 0.7	-7.1%
PC ae C40:5	3.4 ± 0.7	3.7 ± 0.7	-6.7%	4.2 ± 0.8	4.5 ± 1.0	-8.0%
PC ae C40:6	4.8 ± 1.2	5.3 ± 1.3	-10.0%	5.4 ± 1.2	6.2 ± 1.8	-13.5%
PC ae C42:0	0.51 ± 0.1	0.53 ± 0.1	-3.7%	0.58 ± 0.2	0.61 ± 0.2	-4.8%
PC ae C42:1	0.37 ± 0.1	0.38 ± 0.1	-2.6%	0.49 ± 0.1	0.53 ± 0.1	-8.4%
PC ae C42:2	0.6 ± 0.1	0.7 ± 0.2	-7.6%	0.8 ± 0.2	0.9 ± 0.3	-11.4%
PC ae C42:3	0.8 ± 0.2	0.9 ± 0.2	-10.2%	0.9 ± 0.3	0.99 ± 0.3	-9.3%
PC ae C42:4	1.0 ± 0.2	1.1 ± 0.3	-10.8%	1.2 ± 0.3	1.3 ± 0.3	-6.4%
PC ae C42:5	2.2 ± 0.5	2.5 ± 0.5	-8.9%	2.1 ± 0.5	2.3 ± 0.5	-6.1%
PC ae C44:3	0.11 ± 0.03	0.12 ± 0.03	-7.4%	0.18 ± 0.04	0.19 ± 0.05	-7.9%
PC ae C44:4	0.4 ± 0.1	0.5 ± 0.1	-10.8%	0.66 ± 0.2	0.70 ± 0.2	-5.7%
PC ae C44:5	2.0 ± 0.6	2.2 ± 0.6	-6.7%	2.04 ± 0.6	2.13 ± 0.6	-4.2%
PC ae C44:6	1.3 ± 0.4	1.4 ± 0.4	-9.4%	1.3 ± 0.4	1.4 ± 0.4	-6.2%
**lysophosphatidylcholines**						
lysoPC a C16:0	98.1 ± 19.8	89.3 ± 18.5	9.8%	85.6 ± 16.7	80 ± 18	6.8%
lysoPC a C16:1	2.9 ± 1.1	2.8 ± 0.9	4.7%	2.8 ± 1.1	2.7 ± 0.9	4.2%
lysoPC a C17:0	1.68 ± 0.5	1.73 ± 0.5	-2.9%	1.5 ± 0.5	1.6 ± 0.5	-4.5%
lysoPC a C18:0	26.6 ± 5.9	25.1 ± 6.2	5.8%	24 ± 5.4	24 ± 5.9	-0.1%
lysoPC a C18:1	20.3 ± 5.9	17.9 ± 5.2	13.8%	18.6 ± 5.2	16.8 ± 5.4	10.2%
lysoPC a C18:2	29.4 ± 10.4	25 ± 8.9	17.4%	27.2 ± 9.1	24 ± 8.6	12.2%
lysoPC a C20:3	2.5 ± 0.7	2.2 ± 0.6	15.1%	2.9 ± 0.8	2.7 ± 0.7	7.1%
lysoPC a C20:4	7.4 ± 2.3	6.1 ± 1.8	20.5%	7.2 ± 2.2	6.4 ± 1.9	11.7%

	KORA F4			KORA F3		
metabolites	male (µmol/l)	female (µmol/l)	Δ (%)	male (µmol/l)	female (µmol/l)	Δ (%)
**sphingomyelins**						
SM (OH) C14:1	5.5 ± 1.4	6.7 ± 1.6	-17.5%	5.4 ± 1.5	6.8 ± 1.9	-19.9%
SM (OH) C16:1	3.0 ± 0.7	3.6 ± 0.9	-17.2%	3.4 ± 0.9	4.2 ± 1.1	-19.9%
SM (OH) C22:1	12.3 ± 2.7	14.2 ± 2.9	-13.4%	13.4 ± 3	16.1 ± 3.6	-16.8%
SM (OH) C22:2	10.0 ± 2.3	12.6 ± 2.7	-20.8%	11.5 ± 2.6	14.5 ± 3.3	-20.7%
SM (OH) C24:1	1.3 ± 0.3	1.4 ± 0.3	-9.4%	2.2 ± 0.5	2.6 ± 0.6	-13.3%
SM C16:0	100.7 ± 16.7	109.1 ± 16.6	-7.7%	91.2 ± 17.6	101.6 ± 21.4	-10.2%
SM C16:1	14.5 ± 2.5	17.1 ± 3.1	-15.2%	13.8 ± 2.7	16.9 ± 3.3	-18.2%
SM C18:0	21.7 ± 4.3	24.4 ± 4.8	-10.9%	24.4 ± 4.9	28.6 ± 6.1	-14.6%
SM C18:1	10.1 ± 2.1	12.3 ± 2.7	-18.0%	9.8 ± 2.3	12.4 ± 2.9	-21.3%
SM C20:2	0.3 ± 0.1	0.4 ± 0.1	-24.5%	1.7 ± 0.6	1.99 ± 0.6	-15.8%
SM C24:0	21.3 ± 4.4	21.6 ± 4.2	-1.5%	24.5 ± 4.7	26.6 ± 5.6	-7.6%
SM C24:1	50.6 ± 9.8	52.9 ± 9.7	-4.4%	70.9 ± 13.4	79 ± 14.6	-10.3%
SM C26:1	0.4 ± 0.1	0.4 ± 0.1	-2.5%	0.8 ± 0.2	0.9 ± 0.2	-7.5%
**hexoses**						
H1	5369.6 ± 1092	5002.1 ± 879	7.3%	6280.5 ± 1648.5	6056.7 ± 1571.3	3.7%
